# Process-Directed Self-Assembly of Copolymer Blends: I. Micro- and Macrophase Separation

**DOI:** 10.1021/acs.macromol.5c02090

**Published:** 2025-10-24

**Authors:** Jiayu Xie, Marcus Müller

**Affiliations:** Institute for Theoretical Physics, 9375 Georg-August University of Göttingen, 37077 Göttingen, Germany

## Abstract

The phase behavior of binary diblock copolymers features a complex interplay between microphase and macrophase separation. Using a combination of self-consistent field-theory (SCFT) and single-chain-in-mean-field (SCMF) simulations, we investigate the self-assembled morphologies of binary blends composed of two linear diblock copolymers, A_1_B_1_ and A_2_B_2_, both in equilibrium and under processing conditions such as sudden quenching and gradual annealing. We focus on cylinder- and lamella-forming copolymers, where the equilibrium phase diagrams exhibit wide macrophase-separation channels when the length asymmetry between short A_1_B_1_ and long A_2_B_2_ copolymers becomes sufficiently large. Notably, our particle-based simulations uncover a strong dependence of the resulting morphology on the processing pathway used to reach the same thermodynamic state point within the macrophase-separation channel. Quenching produces a homogeneous mixture of A_1_B_1_ and A_2_B_2_ copolymers, a narrow cylinder-size distribution, and stronger hexagonal order, whereas annealing induces local demixing of the two copolymers, yielding a bimodal size distribution and weaker hexagonal order. This process-dependent behavior originates from the complex free-energy landscape of the blends. Overall, our study provides insights into the structure–processing–property relationships in block copolymer systems and contributes to the development of rational processing strategies for targeted nanostructures.

## Graphical abstract

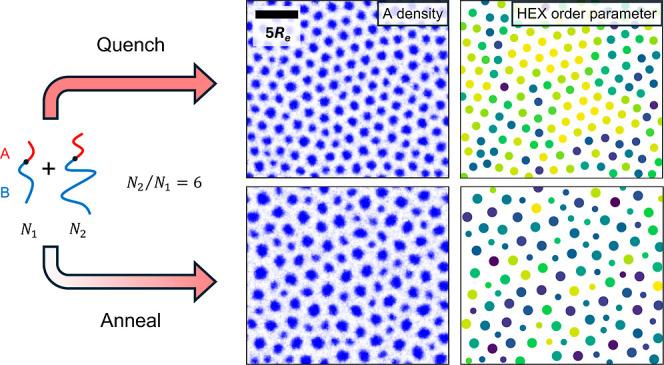

## Introduction

Block copolymers have attracted considerable research attention as they provide a bottom-up route to fabricate nanostructures through self-assembly. Their ability to spontaneously develop long-range order arises from an intrinsic molecular frustration: the chemically distinct blocks tend to phase separate to minimize unfavorable contacts, yet macroscopic phase separation is precluded by the covalent bonds linking the blocks. Consequently, microphase separation occurs, yielding an equilibrium periodicity typically on the molecular scale of 10–100 nm.^1^ The resulting well-ordered nanostructures are useful in a broad range of applications.^2–7^

Block copolymer phase behavior arises from a competition between the energetic cost of forming AB interfaces and the entropic penalty of nonuniform chain stretching required to fill space. This competition generates a complex free-energy landscape with global and local minima corresponding to thermodynamically stable and metastable structures. For the prototypical AB diblock copolymer, this landscape is well captured by three coarse-grained molecular parameters:^8–11^ *χN*, the product of the Flory–Huggins interaction parameter (χ) and the degree of polymerization (*N*), which measures block incompatibility; the block composition, defined by the A-block volume fraction (*f*_A_); and the conformational asymmetry arising from differences in block stiffness.^12,13^ Increasing block-composition asymmetry induces spontaneous curvature at the AB interface, thereby driving transitions from morphologies with flat interfaces (i.e., lamellae) to those with higher interfacial curvature (e.g., networks, cylinders, and spheres).

Beyond single-component copolymer systems, polymer blends containing block copolymers have drawn considerable interest in recent years due to their novel phase behavior.^14–18^ In polymer blends, the coassembly of different polymeric species opens the door to new nanostructures that are absent in the individual components.^19–25^ Even with relatively simple blend formulations, such as binary diblock copolymer/homopolymer blends^26–32^ or diblock copolymer/diblock copolymer blends,^33–40^ a plethora of unconventional mesophases can become accessible. Furthermore, polymer blends provide a cost-effective platform to precisely control the feature size of the self-assembled microdomains, which is valuable in applications including nanolithography^41,42^ and porous membrane fabrication.^43–46^

An intrinsic property of polymer blends is their propensity for macrophase separation, where distinct macromolecular species demix on a macroscopic length scale. For example, when the homopolymer content is high, diblock copolymer/homopolymer blends typically coexist as a homopolymer-rich disordered phase and a copolymer-rich ordered or disordered phase.^14–16,35,47–49^ For diblock/diblock mixtures, although miscibility is generally enhanced by localizing the junctions of both copolymers at the domain interfaces (cosurfactant effect),^50^ the system can still exhibit a wide miscibility gap in blend composition when the length disparity between the two copolymers is large.^51–55^ In macrophase-separated diblock/diblock mixtures, phases can be either disordered or microphase-separated depending on the strength of block–block incompatibility. The interplay between micro- and macrophase separation gives rise to a complex free-energy landscape, making morphological control in such blends particularly challenging.

Besides the equilibrium morphology of block copolymer blends, it is also crucial to understand the nonequilibrium structures and the kinetic pathways that connect them. Owing to the topographical complexity of the free-energy landscape, the system harbors numerous metastable states and can readily become trapped in one of them.^56^ Accordingly, a central practical question is how different processing conditions used to induce ordering in block copolymer blends influence the resulting morphology.^56^ While the equilibrium phase behavior of block copolymer blends has been extensively studied,^23,53–55^ the influence of processing conditions on micro- and macrophase separation, and hence on the resulting self-assembled morphology, remains only partially understood.

In this study, we investigate the process-directed self-assembly of binary diblock copolymer blends using self-consistent field-theory (SCFT)^10,11,57–60^ in combination with particle-based simulations. Focusing on ordered structures with one- and two-dimensional density variations, we first compute the equilibrium phase diagrams of the blends via SCFT. We then explore the parameter space where macrophase separation is thermodynamically expected to occur, examining the kinetics of structure formation from the disordered state to ordered structures and the resulting metastable, nonequilibrium morphologies under two typical processing conditions: sudden thermal quenching and gradual cooling (annealing). To this end, we employ a soft, coarse-grained model in conjunction with the SCMF algorithm to capture both collective density evolution and single-chain dynamics.^61–63^ Our simulations demonstrate that the resulting blend morphologies depend critically on the processing protocol, a consequence of the intricate topography of the free-energy landscape. Our findings provide valuable insights into process-directed self-assembly of block copolymer blends and advance strategies for controlling self-assembled morphologies on the nanoscale through processing pathways. This process dependence is further exemplified by the evaporation-induced self-assembly (EISA) and nonsolvent-induced phase separation (NIPS) methods in the follow-up study, Paper II.^64^

## Theory and Simulation Methods

To develop a comprehensive understanding of both equilibrium and nonequilibrium behavior, we combine theoretical and simulation approaches. Specifically, we employ SCFT^10,11,57–60^ to construct the equilibrium phase diagrams and apply random-phase approximation (RPA)^8,65,66^ to perform a linear stability analysis of the disordered state, thereby gaining insight into the spinodal behavior of the system. To probe kinetic pathways of structure formation and nonequilibrium metastable states, we further carry out SCMF simulations^62^ of a soft, highly coarse-grained particle-based model.

SCFT and RPA for the binary A_1_B_1_/A_2_B_2_ diblock copolymer blends are formulated based on the discrete Gaussian chain model. The blocks forming the first, short copolymer and the second, long copolymer are labeled by the subscript “1” and “2”, respectively. In the discrete Gaussian chain model, each polymer is composed of coarse-grained beads connected by harmonic bonds. A detailed description of SCFT and RPA is provided in section Self-Consistent Field Theory (SCFT) and Section Random-Phase Approximation (RPA) of the Supporting Information. With the chain-contour discretization of the A_1_B_1_ copolymer fixed at *N*_1_ = 64 throughout this work, the key parameters characterizing the binary blends are the A-block composition of the two copolymers (*f*_1_ and *f*_2_), the chain-length ratio (γ_2_ = *N*_2_/*N*_1_), interaction strength (χ_AB_ *N*_1_), and blend composition quantified by the volume fraction of the A_2_B_2_ copolymers ( ${\mathrm{\phi ̅}}_{2}$ ).

To investigate the dynamical and nonequilibrium behavior of the blends, we perform Monte Carlo (MC) simulations using the SCMF algorithm.^62^ In SCMF simulations, soft nonbonded interactions are decoupled through the introduction of quasi-instantaneous fields, which enables efficient simulations of large system sizes and long time scales.^62^ In our simulations, the copolymers are modeled as chains of soft, highly coarse-grained segments connected by harmonic bonds, identical to the molecular model employed in SCFT and RPA. With this model, simulating polymeric systems with a realistic value of invariant degree of polymerization, $\sqrt{\mathrm{N̅}}\equiv \rho_{0}R_{e}^{3}/N_{1}∼10^{2}$ , becomes tractable.^67^ In the current study, $\sqrt{\mathrm{N̅}}=380$ is used in all simulations.

A simulation configuration is specified by the positions of all coarse-grained beads, {**r**_*it*_}, with *i* and *t* running over all chains and segments within a chain, respectively. A set of concentration fields {ϕ_α_(**c**)} in the cell **c** of a three-dimensional (3D) collocation grid with spacing Δ*L* = *R*_e_/10 is defined for each configuration^62,63^

1 $${\mathrm{\phi ̂}}_{\alpha }\left(c\right)=\frac{1}{\rho_{0}\Delta L^{3}}\sum_{it}\delta_{\alpha_{i}\left(t\right),\alpha }\Pi_{c}\left(r_{it}\right)$$

In eq 1, the sum runs over all segments of type α. Π_**c**_ is the characteristic function of the grid cell, **c**, i.e., it takes the value 1 if its argument **r** is within the cell **c**, and 0 otherwise.

With the concentration fields evaluated from the particle coordinates, the pairwise, nonbonded interactions are defined as^62,63^

2 HnbN̅kBT=ΔL3Re3∑c{∑α,β(≠α)χαβN12ϕ̂α(c)ϕ̂β(c)+κN12[∑αϕ̂α(c)−1]2}

where the first term represents the pairwise interactions between distinct types of beads, and the second term suppresses deviations of the total concentration from unity. For all simulations in this work, we fix the parameter κ*N*_1_ = 85, which is proportional to the inverse, isothermal compressibility.

All simulations are performed using the open-source software SOft coarse grained MC Acceleration (SOMA).^63^ We bias the local MC displacements of the beads using the strong bonded forces,^68^ resulting in Rouse-like dynamics.^69^ The time unit, τ_*R*_, is set by the time required for a linear, noninteracting polymer with *N*_1_ segments to diffuse its end-to-end distance (*R*_e_), which is measured to be 34,050 Monte Carlo steps (MCS) for *N*_1_ = 64.

As the scope of this study is restricted to one-dimensional (1D) and two-dimensional (2D) ordered phases, we reduce the computational cost by considering a quasi-2D simulation box with dimensions 24 × 21 × 1*R*_e_^3^. Periodic boundary conditions are applied in all directions.

## Results and Discussion

### Equilibrium Phase Diagrams

The equilibrium phase behavior of binary A_1_B_1_/A_2_B_2_ blends has been investigated previously using SCFT.^33,35,53–55^ In the present study, rather than examining the full parameter space, we restrict our attention to a representative case in which the short A_1_B_1_ copolymer adopts a cylindrical morphology with *f*_1_ = 5/16, while the long A_2_B_2_ copolymer exhibits either a cylindrical (*f*_2_ = 5/16) or a lamellar (*f*_2_ = 1/2) morphology. We further restrict our analysis to 1D and 2D ordered morphologies—lamellae (LAM) and hexagonally packed cylinders (HEX)—together with the disordered (DIS) phase. This setup enables a direct comparison between SCFT phase diagrams and subsequent quasi-2D SCMF simulations, thereby providing the equilibrium reference.

We begin by presenting a ${\mathrm{\phi ̅}}_{2}$ −γ_2_ phase diagram constructed with *f*_1_ = *f*_2_ = 5/16 and χ_AB_ *N*_1_ = 17.5 using SCFT in Figure 1a. For neat A_1_B_1_ copolymers, χ_AB_ *N*_1_ = 17.5 corresponds to a modest degree of segregation. However, the addition of the long A_2_B_2_ copolymers renders the overall segregation degree much stronger. As shown in Figure 1a, the two copolymers are well mixed, forming a single HEX morphology across the entire blend-composition range for small γ_2_ values, e.g., 1 ≤ γ_2_ ≤ 2.25. In Figure 1b, larger γ_2_ values ranging from 4 to 6 are investigated, while all other parameters are kept the same as in Figure 1a. It is observed that the blends remain miscible over the entire ${\mathrm{\phi ̅}}_{2}$ range up to γ_2_ ≈ 5. At γ_2_ = 6, a miscibility gap emerges near ${\mathrm{\phi ̅}}_{2}=0.25$ , thereby dividing the HEX stability window into two distinct regions: one at lower ${\mathrm{\phi ̅}}_{2}$ (corresponding to fewer long copolymers), associated with a small-period (abbreviated “small” hereafter) HEX phase, and the other at higher ${\mathrm{\phi ̅}}_{2}$ , associated with a large-period HEX phase (hereafter referred to as “large HEX phase”). Within the gap, denoted 2ϕ, the A_1_B_1_ and A_2_B_2_ copolymers macrophase separate, resulting in the coexistence of small and large HEX phases. The increased tendency of the blends to undergo macrophase separation into a large phase and a small phase, as γ_2_ increases, is intuitive: the greater the disparity in chain lengths, the more difficult it becomes to incorporate them into a single ordered phase, due to the pronounced mismatch in their length scales.

**Figure 1:**
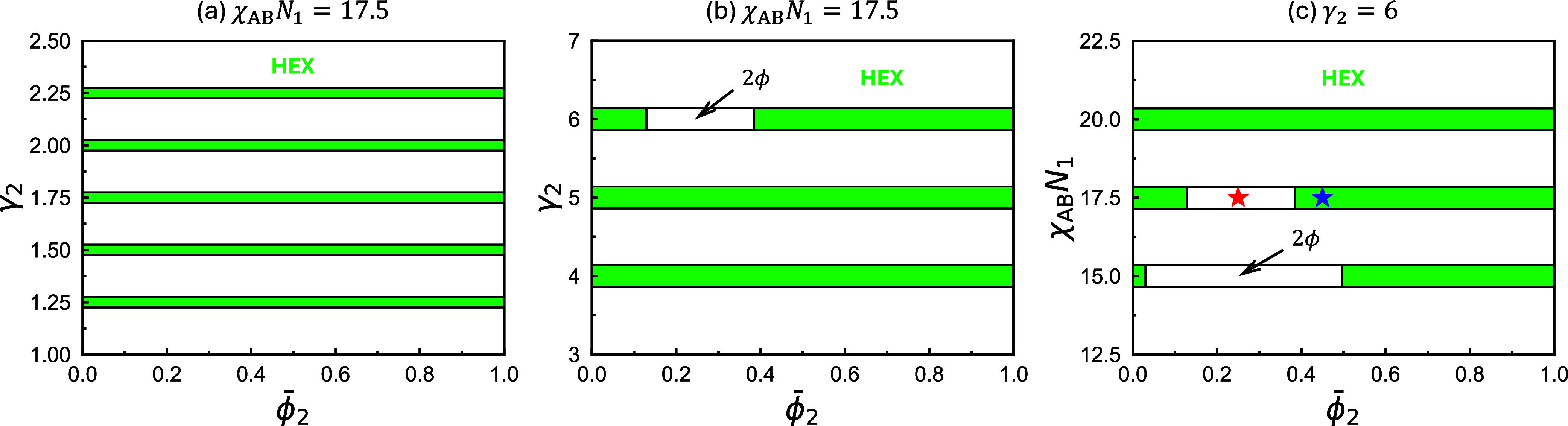
Equilibrium phase diagrams constructed using SCFT for binary blends of diblock copolymers with A-block fractions *f*_1_ = *f*_2_ = 5/16, at different chain-length ratios γ_2_ = *N*_2_/*N*_1_ and incompatibilities χ_AB_ *N*_1_. All phase diagrams in this work are constructed by considering only 1D (LAM) and 2D (HEX) ordered morphologies, together with the DIS phase. The relevant parameters used to construct each diagram are indicated above their respective plots. Star symbols mark the points at ${\mathrm{\phi ̅}}_{2}=0.25$ (red) and 0.45 (blue). A more detailed phase diagram, constructed with the same parameters as in (c), is shown in Figure 10a.

**Figure 2:**
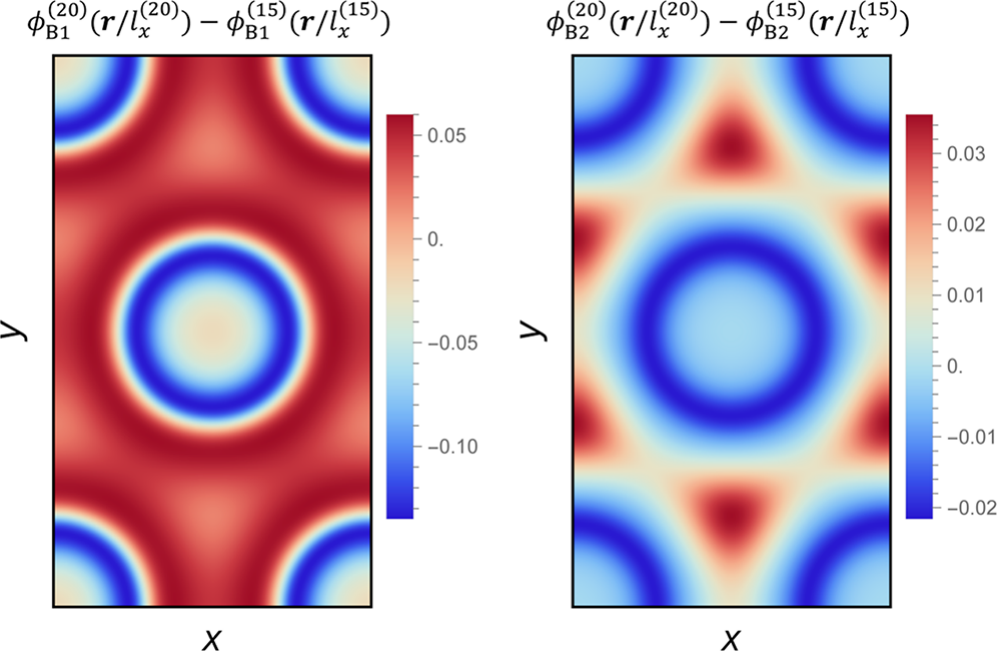
B-block density differences between the two HEX phases at the state points χ_AB_ *N*_1_ = 20 and 15 for *f*_1_ = *f*_2_ = 5/16, ${\mathrm{\phi ̅}}_{2}=0.2$ , and γ_2_ = 6, cf. Figure 1c. The data at χ_AB_ *N*_1_ = 15 correspond to a metastable, homogeneous HEX phase. Panel (a) corresponds to A_1_B_1_ copolymers, and panel (b) corresponds to A_2_B_2_ copolymers. The unit cell dimensions at different χ_AB_ *N*_1_ are normalized by dividing the respective *l*_*x*_ before subtraction, such that *x* spans from 0 to 1 and *y* spans from 0 to $\sqrt{3}$ . The parenthesized superscript for any variable indicates its corresponding χ_AB_ *N*_1_. At χ_AB_ *N*_1_ = 20 and 15, *l*_*x*_ = 2.13*R*_e_ and 2.06*R*_e_, respectively.

**Figure 3:**
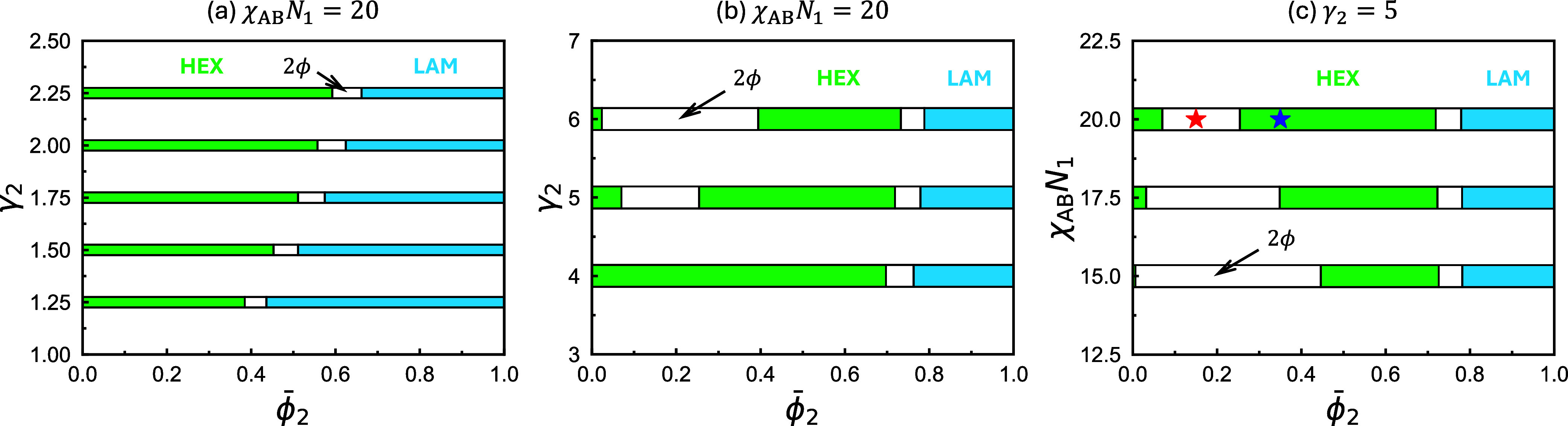
Equilibrium phase diagrams constructed using SCFT for binary blends of diblock copolymers with A-block fractions *f*_1_ = 5/16 and *f*_2_ = 1/2, at different chain-length ratios γ_2_ = *N*_2_/*N*_1_ and incompatibilities χ_AB_ *N*_1_. The relevant parameters used to construct each diagram are indicated above their respective plots. Star symbols mark the state points ${\mathrm{\phi ̅}}_{2}=0.15$ (red) and 0.35 (blue). A more detailed phase diagram, constructed with the same parameters as in (c), is shown in Figure 10b.

**Figure 4:**
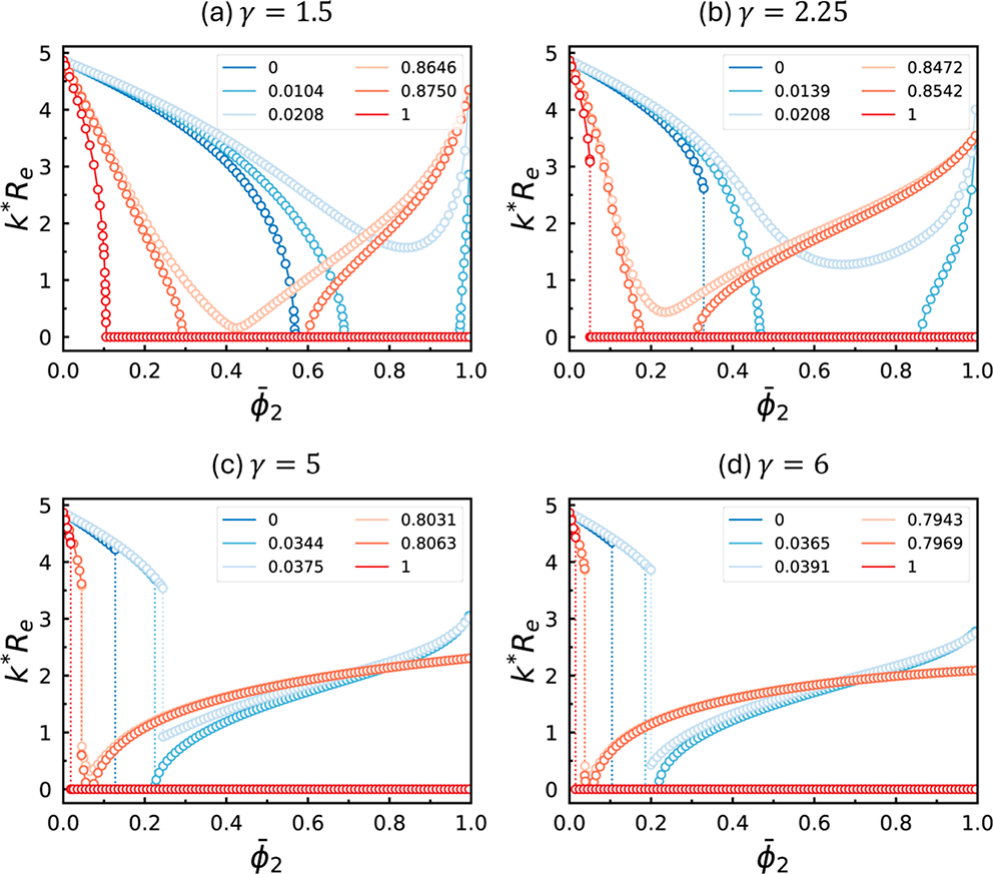
Wavevectors, k*, at which the homogeneous structure first becomes unstable—i.e., at the RPA-predicted spinodal—as a function of ${\mathrm{\phi ̅}}_{2}$ for several representative values of *f*_2_, for blends with γ_2_ = (a) 1.5, (b) 2.25, (c) 5, and (d) 6. In all cases, *f*_1_ = 5/16 is fixed. The *f*_2_ value corresponding to each curve is indicated in the legend. Vertical dotted lines mark discontinuous changes in k*. For each γ_2_, the curves are grouped into two categories, shown in blue and red tones. The blue (red) group contains one curve at *f*_2_ = 0 (*f*_2_ = 1) and two additional curves just below and above the critical *f*_2_ value, beyond (below) which the k* = 0 channel across the blend composition disappears.

**Figure 5:**
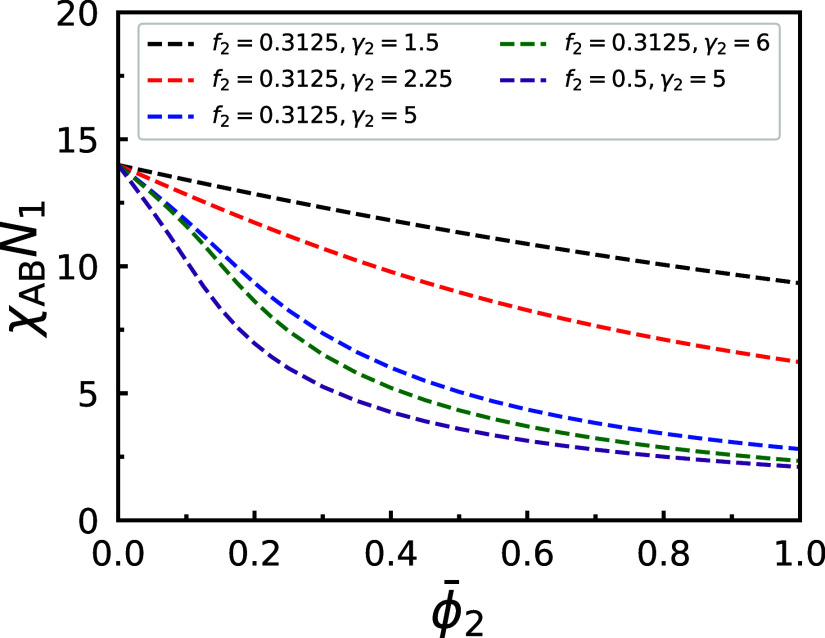
RPA-predicted spinodal curves on the ${\mathrm{\phi ̅}}_{2}$ −χ_AB_ *N*_1_ plane for several representative blends with fixed *f*_1_ = 5/16 and varying combinations of *f*_2_ and γ_2_. The DIS phase is unstable above the spinodal lines, and (meta)­stable below.

**Figure 6:**
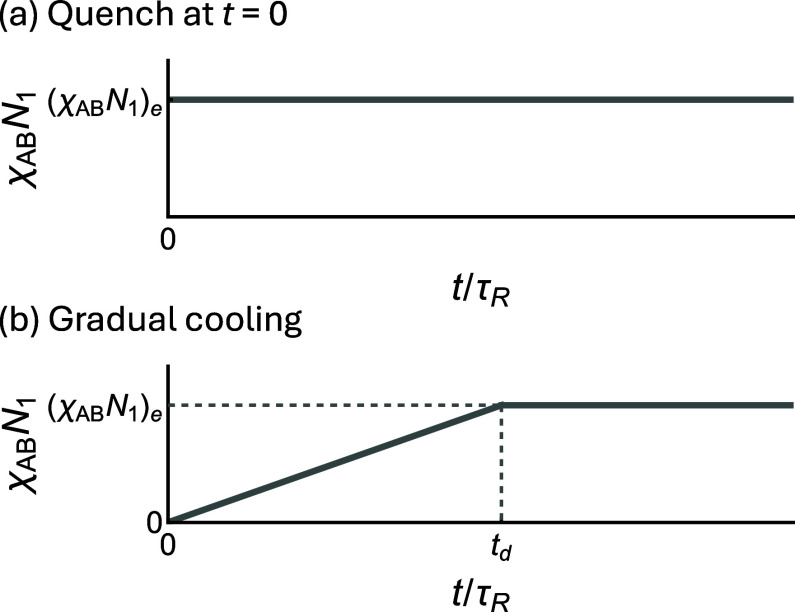
Time evolution of χ_AB_ *N*_1_ for two processing protocols: (a) sudden quenching and (b) gradual cooling (annealing). For quenching, χ_AB_ *N*_1_ changes abruptly from 0 to the target value ${\left(\chi_{AB}N_{1}\right)}_{e}$ at *t* = 0. For annealing, χ_AB_ *N*_1_ increases linearly from 0 at *t* = 0 to the target value ${\left(\chi_{AB}N_{1}\right)}_{e}$ at *t* = *t*_*d*_, and remains fixed at ${\left(\chi_{AB}N_{1}\right)}_{e}$ thereafter.

**Figure 7:**
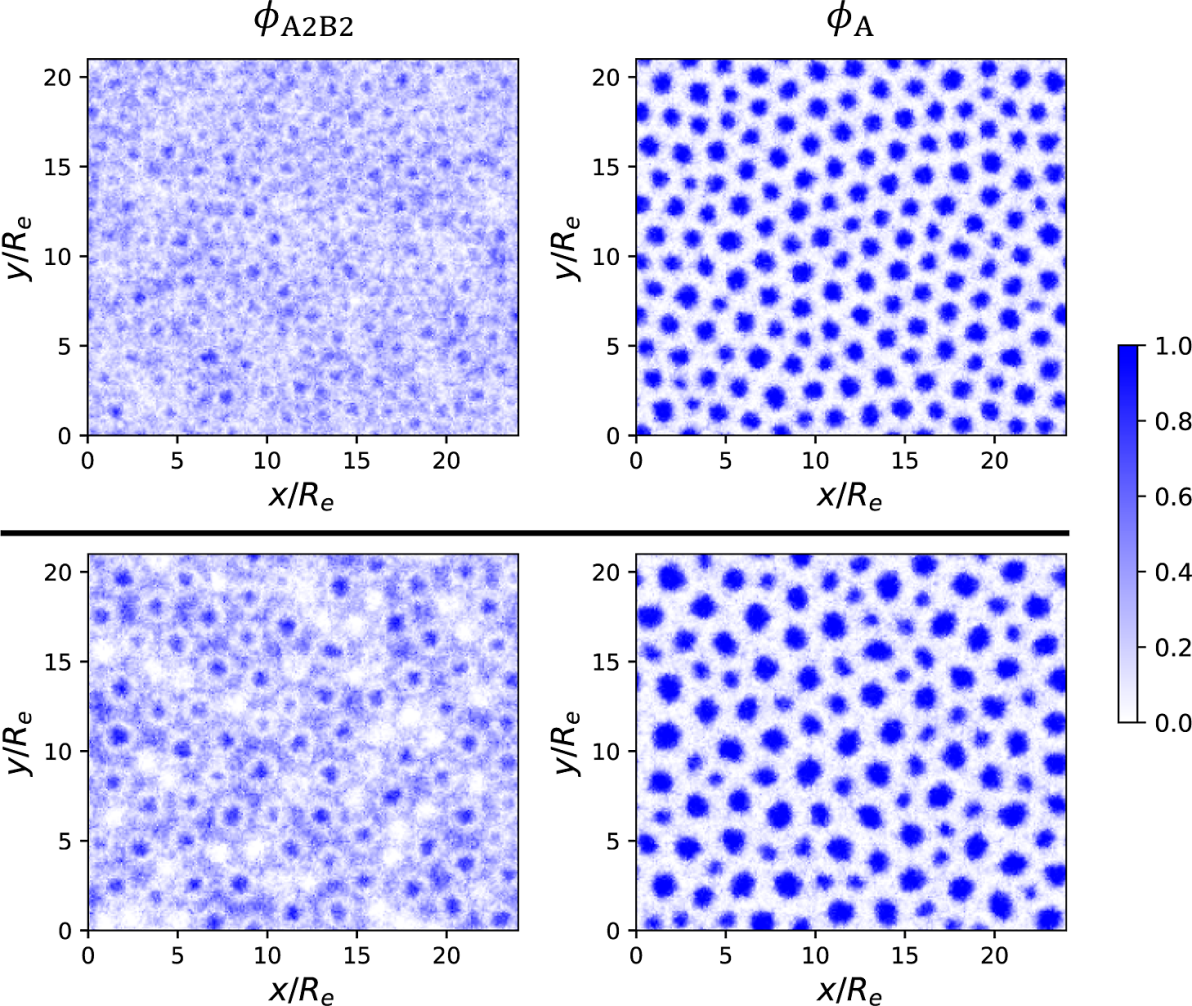
Final SCMF simulation snapshots at *t* = 40τ_*R*_ showing the density distributions of A_2_B_2_ copolymers (left column), and the total A blocks (right column), for the quenched system (top row) and the annealed system (bottom row). Both simulations target the state point ( ${\mathrm{\phi ̅}}_{2}=0.25$ , χ_AB_ *N*_1_ = 17.5, *f*_1_ = *f*_2_ = 5/16, γ_2_ = 6) indicated by the red star in Figure 1c. The density of each component is rescaled such that the total density sums to unity at each spatial position, yielding normalized density profiles that represent the local composition. This normalization is applied to all density plots throughout this work, resulting in a consistent color bar ranging from 0 to 1.

**Figure 8:**
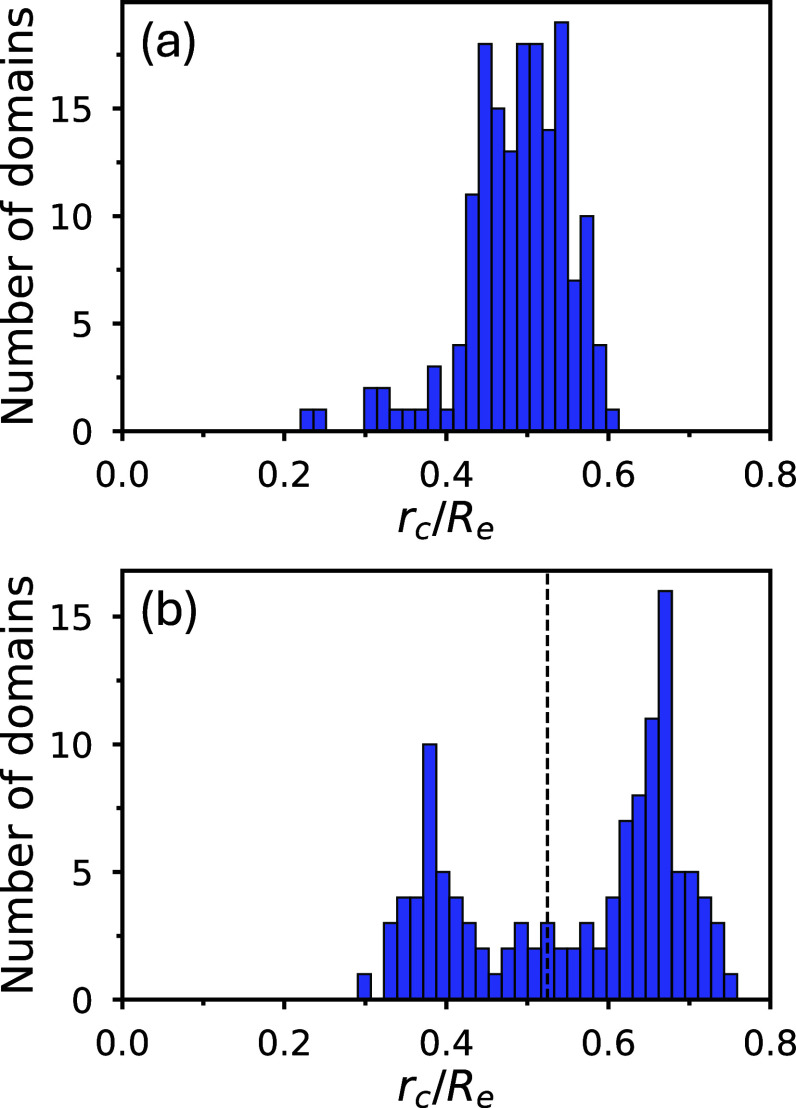
Histograms of A-rich domain radii measured from simulation snapshots shown in the (a) top and (b) bottom panels of Figure 7. In panel (b), the distribution exhibits a pronounced bimodal feature, with the vertical dashed line indicating the midpoint between the left and right peaks.

**Figure 9:**
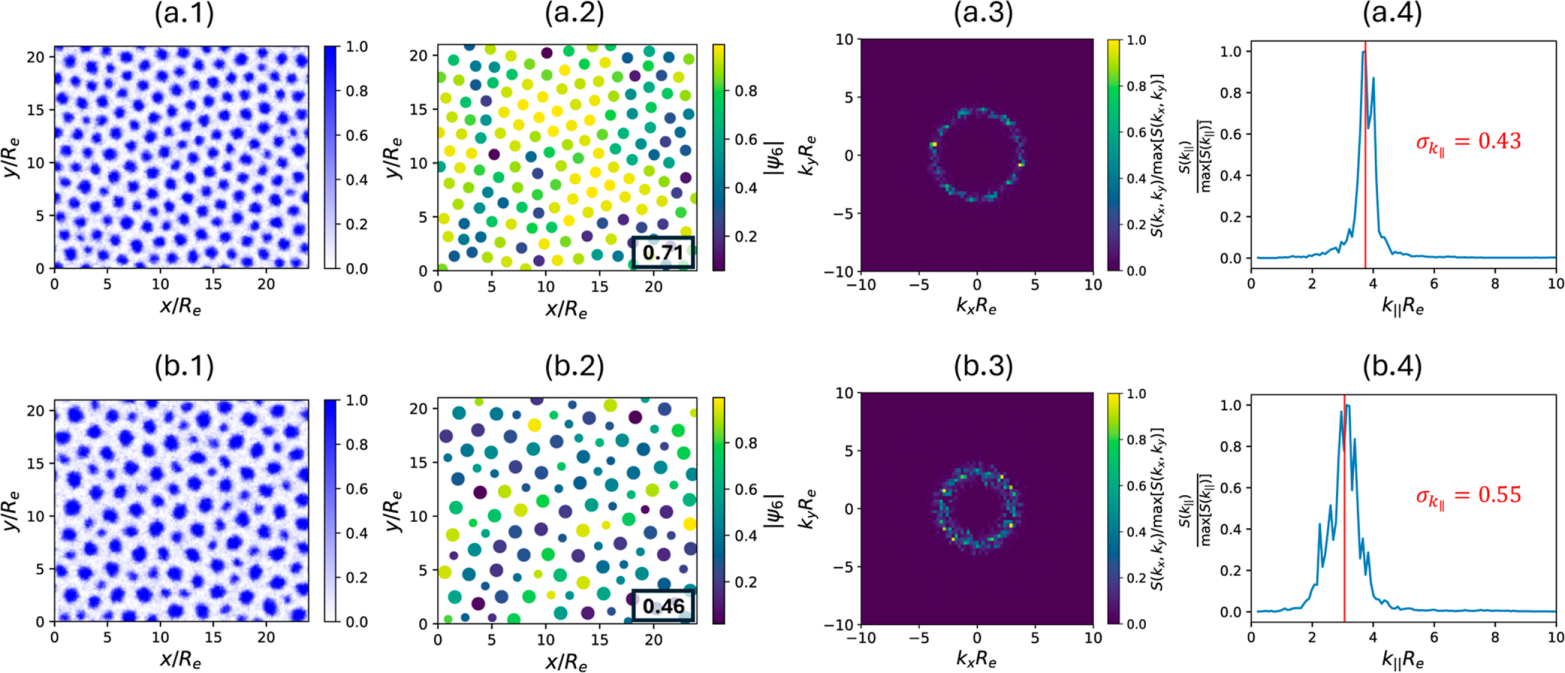
Normalized A-block density profiles (x.1), hexatic order parameter |ψ_6_| (x.2), 2D structure factor *S*(*k*_*x*_, *k*_*y*_) (x.3), and azimuthally averaged structure factor *S*(*k*_∥_) (x.4), with *x* = a and b corresponding to the final snapshots of the quenching and annealing simulations in Figure 7, respectively. In panel (x.2), the average value ⟨|ψ_6_|⟩ is indicated at the bottom right corner of each plot. For the annealed system, the domains in (b.2) are divided into small and large groups by comparing their radii with the threshold indicated by the vertical dashed line in Figure 8, and are displayed as small and large circles, respectively. In contrast, for the quenched system, the domains in (a.2) are shown as circles of identical size. In panel (x.4), the vertical red line in each plot indicates the ⟨*k*_∥_⟩, with *σ*_*k*_∥__ in units of *R*_e_ labeled alongside it.

Figure S3 provides additional information on the distributions of the two types of copolymers in the HEX phase. When the HEX phase contains a majority of short A_1_B_1_ chains, although their density is localized at the interface, a substantial amount also distributes away from the interface into both the domain cores and matrix, regardless of the γ_2_ value. In contrast, when the blend contains a majority of long A_2_B_2_ chains, the systems behave differently, depending on the value of γ_2_. For small γ_2_, increasing ${\mathrm{\phi ̅}}_{2}$ from 0.2 to 0.8 primarily results in a nearly position-independent shift of the local densities of the two copolymers, without significantly altering their spatial variations. However, for large γ_2_, the increase in ${\mathrm{\phi ̅}}_{2}$ rapidly increases the size of the domains and period of the lattice, leading to a strong localization of the short copolymers at the interface and a near-complete depletion of them from the domain cores. These observations are illustrated more clearly by the 1D density profiles shown in Figure S4, obtained by tracing the density along the path marked by the white dashed line in Figure S3a.

To evaluate the influence of the incompatibility χ_AB_ *N*_1_, Figure 1c presents a ${\mathrm{\phi ̅}}_{2}$ −χ_AB_ *N*_1_ phase diagram at fixed γ_2_ = 6. As χ_AB_ *N*_1_ increases from 15 to 17.5 and then to 20, the phase coexistence window shrinks and eventually disappears (the HEX + HEX coexistence window closes at a critical point on the ${\mathrm{\phi ̅}}_{2}$ −χ_AB_ *N*_1_ plane as shown later in Figure 10). This indicates that the coexisting short- and long-copolymer–rich microphases[Fn fn1] exhibit an enhanced capacity to accommodate the poor copolymer species, thereby narrowing the miscibility gap and ultimately eliminating the coexistence region at high incompatibility. As the interfacial free energy between A-rich and B-rich domains increases with χ_AB_ *N*_1_, packing frustration becomes more pronounced. This frustration originates from the competition between the tendency of AB interfaces to remain circular—minimizing interfacial energy—and the requirement that B blocks stretch into the interstitial space to maintain a constant total segment density. Blending partially alleviates this frustration: the domain spacing in the blend exceeds that of the pure A_1_B_1_ HEX phase, and the B blocks of the long copolymers preferentially segregate into the interstitial regions, thereby reducing the need for excessive stretching of the shorter A_1_B_1_ chains. The increased segregation of the B blocks of the short copolymers toward the internal AB interfaces, together with the localization of the majority block of the long copolymers in the interstitials, is illustrated in Figure 2. This segregation pattern within the hexagonal unit cell explains the reduced propensity of the blend to undergo macrophase separation with increasing χ_AB_ *N*_1_, as shown in Figure 1c.

**Figure 10:**
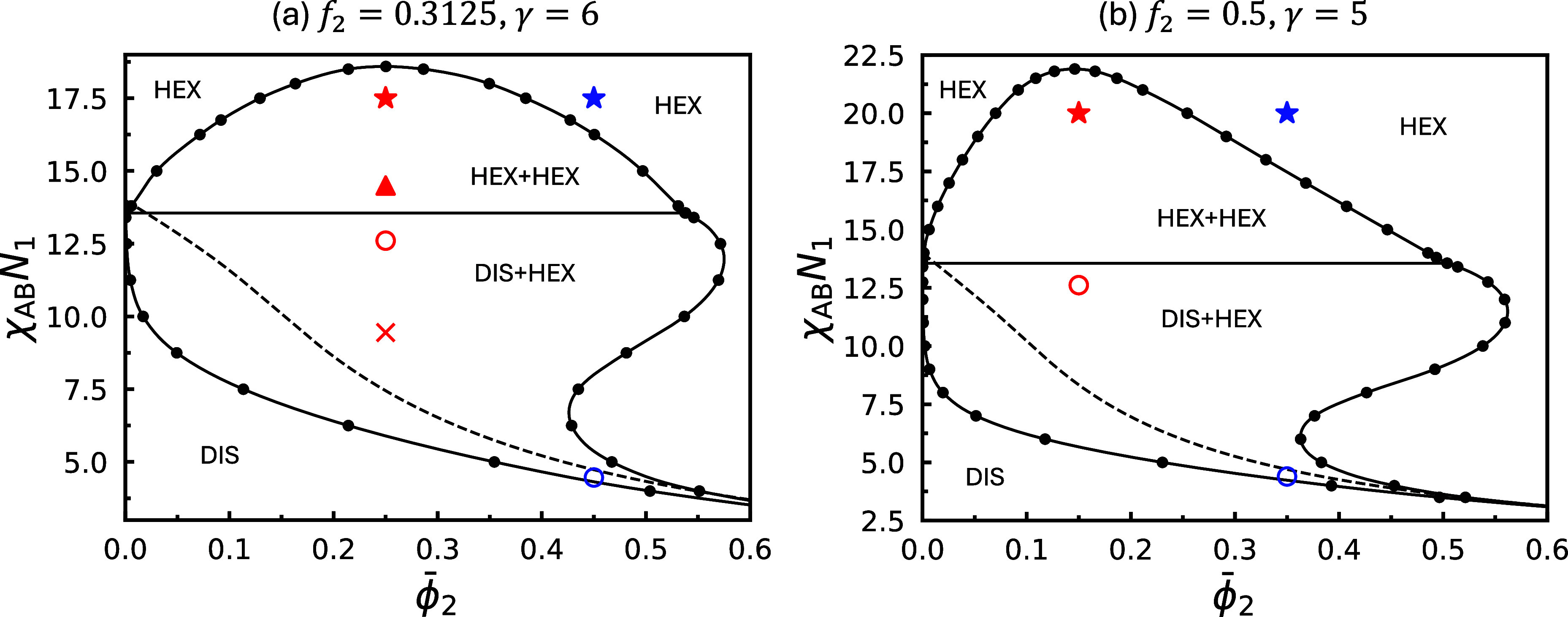
Equilibrium SCFT phase diagrams constructed with the same parameters as those used in (a) Figure 1c (*f*_1_ = *f*_2_ = 5/16 and γ_2_ = 6) and (b) Figure 3c (*f*_1_ = 5/16, *f*_2_ = 1/2 and γ_2_ = 5), but with more finely resolved phase boundaries. The solid, horizontal line represents the triple line, separating the HEX + HEX and DIS + HEX coexistence regions, and the dashed line denotes the spinodal of the DIS structure. Star symbols correspond to those in Figures 1c and 3c, using identical colors. Open circles mark the estimated metastability limit of the homogeneous HEX structure at ${\mathrm{\phi ̅}}_{2}$ = (a) 0.25 or (b) 0.15 (red), and (a) 0.45 or (b) 0.35 (blue). In (a), the red triangle and cross mark the points χ_AB_ *N*_1_ = 14.5 and 9.4469, respectively, at ${\mathrm{\phi ̅}}_{2}=0.25$ .

Figure 3 shows three phase diagrams for blends comprising the same A_1_B_1_ copolymers as in Figure 1 and lamella-forming A_2_B_2_ copolymers with *f*_2_ = 1/2. As in Figure 1, a miscibility gap separating the small- and large-period HEX phases is present at high γ_2_ values. In addition, order–order transitions from HEX to LAM are observed at large ${\mathrm{\phi ̅}}_{2}$ for all γ_2_ values. The concomitant HEX + LAM coexistence region remains narrow across all γ_2_, whereas the HEX + HEX region broadens rapidly with increasing γ_2_ (see Figure 3b). Moreover, Figure 3c shows contrasting trends with varying χ_AB_ *N*_1_: the HEX + HEX coexistence region contracts, consistent with Figure 1c, whereas the HEX + LAM window remains nearly unchanged. The nearly invariant width of the HEX + LAM coexistence window upon varying ${\mathrm{\phi ̅}}_{2}$ and χ_AB_ *N*_1_ suggests a different origin of macrophase separation than that of the HEX + HEX window. Unlike the HEX + HEX coexistence region, which primarily arises from relieving chain-packing frustration between copolymers of drastically different length scales, the narrow HEX + LAM region emerges from a free-energy gain associated with fractionation into phases of different symmetries but not necessarily significantly different length scales.

### Process-dependent Nonequilibrium Morphologies: Micro- and Macrophase Separation

Processing here refers to a change in the thermodynamic state point. We investigate the kinetics of structure formation from an initially disordered state (DIS) to a microphase-separated target state, varying the incompatibility χ_AB_ *N*_1_ in time while keeping the molecular characteristics of the copolymer blend—namely, the chain-length ratio γ_2_, the overall composition ${\mathrm{\phi ̅}}_{2}$ , and the segmental symmetry—unaltered. In particular, we focus on target states at high γ_2_, where a broad HEX + HEX coexistence region emerges (see Figures 1 and 3). Our goal is to understand how processing conditions dictate the resulting (meta)­stable structure emerging from the interplay between microphase and macrophase separation.

#### RPA Analysis of the Spinodal Curves

Before delving into simulations, it is informative to assess the spinodal behavior by conducting a linear instability analysis using RPA for the disordered state of the binary mixtures. For given set of parameters, the system can undergo instability against composition fluctuations with either a finite or infinite length scale, characterized by a nonzero or zero critical wavevector k* at the spinodal, respectively.

Figure 4 presents the wavevector k* of the unstable mode at the spinodal as a function of ${\mathrm{\phi ̅}}_{2}$ for various representative values of *f*_2_ and γ_2_ with *f*_1_ = 5/16 held fixed. For each curve, any region along the blend composition where k* = 0 indicates an instability with respect to macrophase separation.[Fn fn2]

In the extreme cases, *f*_2_ = 0 or *f*_2_ = 1, the A_2_B_2_ copolymers degenerate to B or A homopolymers, which are known to exhibit large immiscible windows when mixed with AB diblock copolymer.^29^ This trend is confirmed by Figure 4 across all γ_2_ values, where broad blend-composition windows of macrophase instability emerge once ${\mathrm{\phi ̅}}_{2}$ exceeds certain thresholds and persist up to ${\mathrm{\phi ̅}}_{2}=1$ .

As the A-block fraction *f*_2_ of the long copolymer becomes more symmetric—either increasing from 0 or decreasing from 1—the macrophase-instability windows contract and eventually vanish. For fixed *f*_1_ and γ_2_, RPA predicts macrophase instability only near *f*_2_ = 0 or 1; otherwise, the unstable mode always exhibits a finite characteristic length scale. Although the overall range of *f*_2_ associated with macrophase instability expands with increasing γ_2_, it remains relatively narrow even at γ_2_ = 6. In this regime, the blends exhibit exclusively microphase instability across the broad composition window 0.0391 ≲ *f*_2_ ≲ 0.7943. Therefore, based on the RPA predictions in Figure 4, the DIS phase of the binary blends exhibits instabilities only against composition fluctuations with finite wavelengths, i.e. microphase separation, within the parameter range covered by the equilibrium phase diagrams in Figures 1 and 3.

It is also worth noting that, along the curves with *f*_2_ = 0 and 1 for γ_2_ = 2.25 (see Figure 4b) and all *f*_2_ values for γ_2_ ≥ 5 (Figure 4c and d), we observe k* to change discontinuously at certain values of ${\mathrm{\phi ̅}}_{2}$ . This first-order-transition-like behavior in k* indicates a sudden change in the preferred length scale of instability upon changing the blend composition, ${\mathrm{\phi ̅}}_{2}$ , and it has also been discovered in various block copolymer/homopolymer blends.^17,18^

Figure 5 presents the spinodal curves on the ${\mathrm{\phi ̅}}_{2}$ −χ_AB_ *N*_1_ plane for several representative blends with varying combinations of *f*_2_ and γ_2_. In all cases, the spinodals exhibit instabilities only at finite wavelengths, indicated by dashed curves. The DIS phase remains (meta)­stable below these curves, while phase separation via spinodal decomposition occurs when the blends are driven into the region above them through specific processing conditions. As ${\mathrm{\phi ̅}}_{2}$ increases, the corresponding spinodal value of χ_AB_ *N*_1_ decreases, reflecting enhanced segregation strength due to the larger proportion of longer A_2_B_2_ copolymers. At fixed ${\mathrm{\phi ̅}}_{2}$ , a larger γ_2_ and/or a more symmetric *f*_2_ result in a lower spinodal threshold of χ_AB_ *N*_1_.

#### Process-dependent Morphologies: Quench vs Annealing

We now move to particle-based simulations of binary A_1_B_1_/A_2_B_2_ blends with large γ_2_ values under varying processing conditions. We first perform two simulations targeting the same state point marked by the red star ( ${\mathrm{\phi ̅}}_{2}=0.25$ , χ_AB_ *N*_1_ = 17.5) on the phase diagram in Figure 1c with γ_2_ = 6. At *t* = 0, both simulations begin with the disordered state where all polymer chains are homogeneously distributed in the simulation cell, corresponding to χ_AB_ *N*_1_ = 0. Ordering is then induced via two different processes, as illustrated in Figure 6. In the first simulation, we suddenly quench the incompatibility to χ_AB_ *N*_1_ = 17.5 at *t* = 0. In the second simulation, χ_AB_ *N*_1_ is linearly increased from 0 (below the order–disorder-transition (ODT)) at *t* = 0 to 17.5 over a duration of 30τ_*R*_, mimicking a gradual cooling (thermal annealing), and stays at 17.5 thereafter. Both simulations run for a total of 40 τ_*R*_, and their final density distributions are shown in Figure 7.

Interestingly, the final morphologies from these two ordering processes exhibit clear distinctions: The A-rich domains formed via quenching show greater size homogeneity and stronger local HEX order. In contrast, the A-rich domains resulting from annealing exhibit two clearly distinct size groups and lack a well-defined HEX arrangement. We quantify the local hexagonal order by evaluating the hexatic order parameter, |ψ_6_|. For domain *i*, this is computed via

3 $$\left|\psi_{6}\left(i\right)\right|=\left|\frac{1}{N_{i}^{Vor}}\sum_{j\in N_{i}^{Vor}}e^{i6\theta_{ij}}\right|$$

where $N_{i}^{Vor}$ denotes the set of *N*_*i*_^Vor^ Voronoi neighbors of domain *i*, and θ_*ij*_ is the angle of the bond vector from domain *i* to its neighbor *j*. For computing |ψ_6_|, the domain positions are taken as the coordinates of their geometric centers. Additionally, the average value of |ψ_6_(*i*)| across all domains, ⟨|ψ_6_|⟩, is computed as a measure of the overall hexagonal order. The results are shown in Figure 9(x.2) with *x* = a and b for the quenching and annealing simulations, respectively, along with their normalized A-block densities reproduced from Figure 7 in Figure 9(x.1). The quenched system clearly exhibits more high-|ψ_6_| domains, with a higher average hexagonal order ⟨|ψ_6_|⟩ = 0.71, compared to ⟨|ψ_6_|⟩ = 0.46 for the annealed system.

Figure 9 also compares the 2D structure factors (panel (x.3)), *S*(*k*_*x*_, *k*_*y*_), and the angle-averaged 1D structure factors (panel (x.4)), *S*(*k*_∥_) with $k_{∥}=\sqrt{k_{x}^{2}+k_{y}^{2}}$ , of the final A densities in the quenched and annealed systems (panel (x.1)). The *S*(*k*_*x*_, *k*_*y*_) of the annealed system displays a thicker ring than that of the quenched system, indicating a broader distribution of length scales in the morphology. Accordingly, the *S*(*k*_∥_) also shows a broader profile for the annealed system. To quantitatively compare the spread of length scales in the A-block densities between these two cases, we compute the standard deviation of the different Fourier modes

4 $$\sigma_{k_{∥}}=\sqrt{\left\langle {\left(k_{∥}-\left\langle k_{∥}\right\rangle \right)}^{2}\right\rangle },with\;\left\langle k_{∥}\right\rangle =\frac{\sum_{k_{∥}}S\left(k_{∥}\right)k_{∥}}{\sum_{k_{∥}}S\left(k_{∥}\right)}$$

In computing ⟨*k*_∥_⟩, only *k*_∥_ with *S*(*k*_∥_) > 0.01 max­[*S*(*k*_∥_)] is included to exclude the influence of irrelevant high-frequency features with vanishingly small intensities. The resulting ⟨*k*_∥_⟩ for each case is indicated as a vertical red line in the third column of Figure 9, with σ_*k*_∥__ labeled next to it. The morphology obtained from annealing exhibits a higher average length scale (lower ⟨*k*_∥_⟩), and a clearly greater σ_*k*_∥__, compared to the one from quenching.

Figure 8 presents the distribution of A-rich domain radii measured from the simulation snapshots in Figure 7. The detailed postprocessing procedure to denoise the simulation data for determining the cylinder radii is described in the Supporting Information of paper II.^64^ Compared to quenching, annealing results in cylindrical domains with a slightly broader size distribution and a pronounced bimodal feature, consistent with the two distinct size groups observed in Figure 7 (bottom right).

A close inspection of the polymer distributions in Figure 7 also reveals qualitative differences. Consistent with the SCFT prediction (see, e.g., Figures S3 and S4), the longer A_2_B_2_ copolymers are depleted from the AB interfaces in both quenched and annealed systems due to the preferential segregation of the shorter A_1_B_1_ copolymers at these regions. This local segregation is reflected as light rings in the A_2_B_2_ density distributions. In the annealed system, however, there exist additional regions exhibiting a pronounced depletion of A_2_B_2_ chains, identifiable as ringless white patches in the A_2_B_2_ density. This local demixing of A_1_B_1_ and A_2_B_2_ copolymers signals the onset of macrophase separation. Consequently, small cylinders emerge within the A_2_B_2_-depleted regions, whereas significantly larger cylinders form in the complementary A_2_B_2_-rich regions, giving rise to the pronounced cylinder size disparity observed in the annealed system. On the contrary, in the quenched system, the long and short chains are more uniformly distributed throughout space, showing no significant local demixing. This results in more monodisperse cylindrical domains and thus an arrangement with fewer defects compared to the annealed system. It is interesting to observe that two systems brought to the same state point on the phase diagram through different processing conditions exhibit qualitatively different behaviors: microphase separation upon quenching, but (local) macrophase separation upon annealing. Since the SCFT-predicted equilibrium state consists of macrophase-separated large and small cylinders, we conclude that the annealed system reaches a state closer to equilibrium than the quenched system, although it remains far from complete macrophase separation, where A_1_B_1_-rich and A_2_B_2_-rich domains form at a larger, macroscopic scale.

To gain a deeper understanding of the observed process dependence of structure formation, we explore the underlying free-energy landscape by SCFT calculations. Specifically, we seek SCFT solutions for the single, homogeneous (not macrophase-separated) HEX phase within the canonical ensemble, starting from the common final state point of the quenched and annealed systems. From this initial state point—marked by the red star in the phase diagram in Figure 1c (and shown in greater detail in Figure 10a)—we systematically decrease χ_AB_ *N*_1_, following the vertical path downward. We observe a sharp increase in the number of SCFT iterations required for convergence as χ_AB_ *N*_1_ falls below a certain threshold, eventually reaching a point—indicated by the open circle in Figure 10—where no converged solution can be obtained. This estimates the metastability limit of the homogeneous (not macrophase-separated) HEX phase, i.e., the spinodal of the single, spatially modulated HEX structure, where it transitions from metastable to unstable.

With identical parameters as those used in Figure 1c, Figure 10a presents more finely resolved phase boundaries, the spinodal curve of the DIS structure, and the spinodal points of the homogeneous HEX structure at selected ${\mathrm{\phi ̅}}_{2}$ values. When quenching the system from the DIS phase below the ODT to a state point between the spinodal of the DIS structure and that of the homogeneous HEX phase, the system will initially undergo spontaneous microphase separation with a finite wavevector k*R_e_ > 0, as predicted by RPA. However, the resulting incipient spatially modulated structure is not metastable. After the linear instability, the system eventually proceeds to macrophase separation.

Importantly, the distinct morphologies observed in the quenched and annealed systems can be rationalized by the results in Figure 10a (see also section Visualization of System Evolution and Free-Energy Landscapes of the Supporting Information for an illustrative explanation). During annealing, the blend undergoes macrophase separation before χ_AB_ *N*_1_ reaches the spinodal of the homogeneous HEX structure, resulting in a locally demixed, partially macrophase-separated structure at the final state point. In contrast, a direct quench to χ_AB_ *N*_1_ = 17.5 drives the system into a region, where the homogeneous HEX structure is metastable. Such a homogeneous HEX structure is templated by the spinodal instability of the DIS structure (although the length scales differ, vide infra Figure 11). Once the junction points of the diblock copolymers become localized at the internal AB interfaces, macrophase separation is severely hindered. Similar to Ostwald ripening,^70^ the relaxation of loops and bridges in triblock copolymer,^71^ or the relaxation of a micelle size distribution, such a relaxation process may monotonically reduce the collective free energy of the system. However, the transfer of an A block from one cylinder to another is an intrinsically slow process due to a substantial single-chain free-energy barrier, strongly limiting chain dynamics.

**Figure 11:**
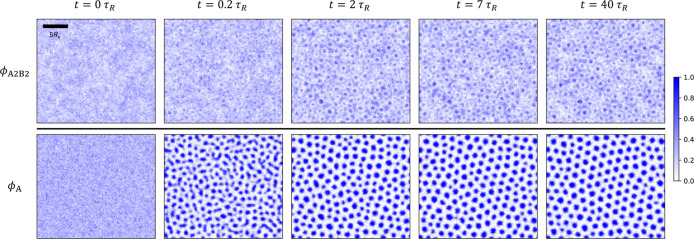
Structure formation upon quenching: quasi-2D SCMF simulation snapshots at representative time steps, showing the time evolution of A_2_B_2_-copolymer (top row), and total-A-block (bottom row) density distributions, for the quenched system targeting the state point marked by the red star in Figure 1c. Axis labels and ticks are the same as in Figure 7 and are omitted here. A scale bar is included in the graph at the top left to indicate the length scale 5*R*_e_.

The distinct mechanisms of structure formation upon quenching and annealing are corroborated by the temporal evolution of the blend components. Figures 11 and 12 present density snapshots at representative time steps for the quenched and annealed systems, respectively.

**Figure 12:**
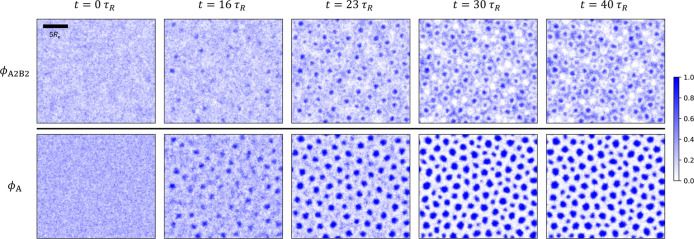
Structure formation upon annealing: quasi-2D SCMF simulation snapshots at representative time steps, showing the time evolution of A_2_B_2_-copolymer (top row), and total-A-block (bottom row) density distributions, for the annealed system targeting the state point marked by the red star in Figure 1c. Axis labels and ticks are the same as in Figure 7 and are omitted here. A scale bar is included in the graph at the top left to indicate the length scale 5*R*_e_.

For the quenched system, spinodal decomposition initiates immediately at *t* ≥ 0, and the A domains evolve into a cylindrical morphology through coarsening already at *t* ≈ 2τ_*R*_. Between 2τ_*R*_ ≲ *t* ≤ 40τ_*R*_, the system slowly relaxes toward the metastable HEX phase. During this period, intrinsically slow interdomain chain exchange and occasional cylinder fusions result in more uniform cylinder sizes and the emergence of HEX order. The enhancement of both local and globally averaged HEX order is illustrated by the time evolution of |ψ_6_| in Figure S5. Apart from the local enrichment of A_1_B_1_ copolymers at the internal AB interfaces, no significant changes in the polymer densities occur, as shown in Figure 11.

In the annealed system, small clusters of A-core micelles are emerging at *t* ≈ 16τ_*R*_, primarily formed by the local enrichment of the longer A_2_B_2_ copolymers within a homogeneous A_1_B_1_-copolymer matrix (Figure 12). At this time, the state point ( $\chi_{AB}N_{1}\left(t\right),{\mathrm{\phi ̅}}_{2}$ ) is located within the DIS + HEX coexistence region of the phase diagram (cf. Figure 10a). As time progresses, the local demixing between the A_1_B_1_ and A_2_B_2_ copolymers becomes increasingly apparent. By *t* ≈ 23τ_*R*_, the state point is approximately located at the triple line, at which the DIS phase coexists with a small and a large HEX phase. After this point, small A_1_B_1_ micelles begin to form. The increasing incompatibility and the formation of additional AB interfaces slows down macrophase separation. For *t* ≥ 30τ_*R*_, the incompatibility, χ_AB_ *N*_1_ = 17.5 remains constant and lies well above the triple line. During this period, the previously developed spatial inhomogeneity in the densities of the long and short copolymers becomes (slightly) more pronounced, finally leading to a partially macrophase-separated state characterized by the coexistence of finite-sized domains of small and large cylinders.

For comparison, we perform two additional simulations using the same quenching and annealing protocols as in Figure 7, but targeting a different state point corresponding to a homogeneous large HEX phase outside the two-phase coexistence region—marked by the blue star ( ${\mathrm{\phi ̅}}_{2}=0.45$ , χ_AB_ *N*_1_ = 17.5) in the phase diagram, cf. Figure 1c. Importantly, for ${\mathrm{\phi ̅}}_{2}=0.45$ , SCFT calculations suggest that the homogeneous HEX structure is metastable for all incompatibilities above the ODT (see Figure 10a).

The resulting final density distributions are presented in Figure 13. In contrast to the previous simulations targeting the state point at ${\mathrm{\phi ̅}}_{2}=0.25$ and χ_AB_ *N*_1_ = 17.5, the present results display more regular copolymer distribution patterns, independent of the processing conditions. The short A_1_B_1_ chains preferentially localize at the internal AB interfaces, forming ring-like depletion zones in the A_2_B_2_ density. Notably, unlike the bottom-middle panel in Figure 7, no systematic formation of ringless white patches is observed during annealing. This indicates that, at this state point, both processing protocols lead to uniform microphase separation, although their average cylinder radii, *r*_*c*_, markedly differ from each other: *r*_*c*_ = 0.58*R*_e_ (quench) and *r*_*c*_ = 0.76*R*_e_ (anneal), as well as from the equilibrium value *r*_*c*_ = 0.81*R*_e_ predicted by SCFT. The differences in structural size arising from distinct processing conditions are discussed in detail in Paper II.^64^ ^,^ [Fn fn3]

**Figure 13:**
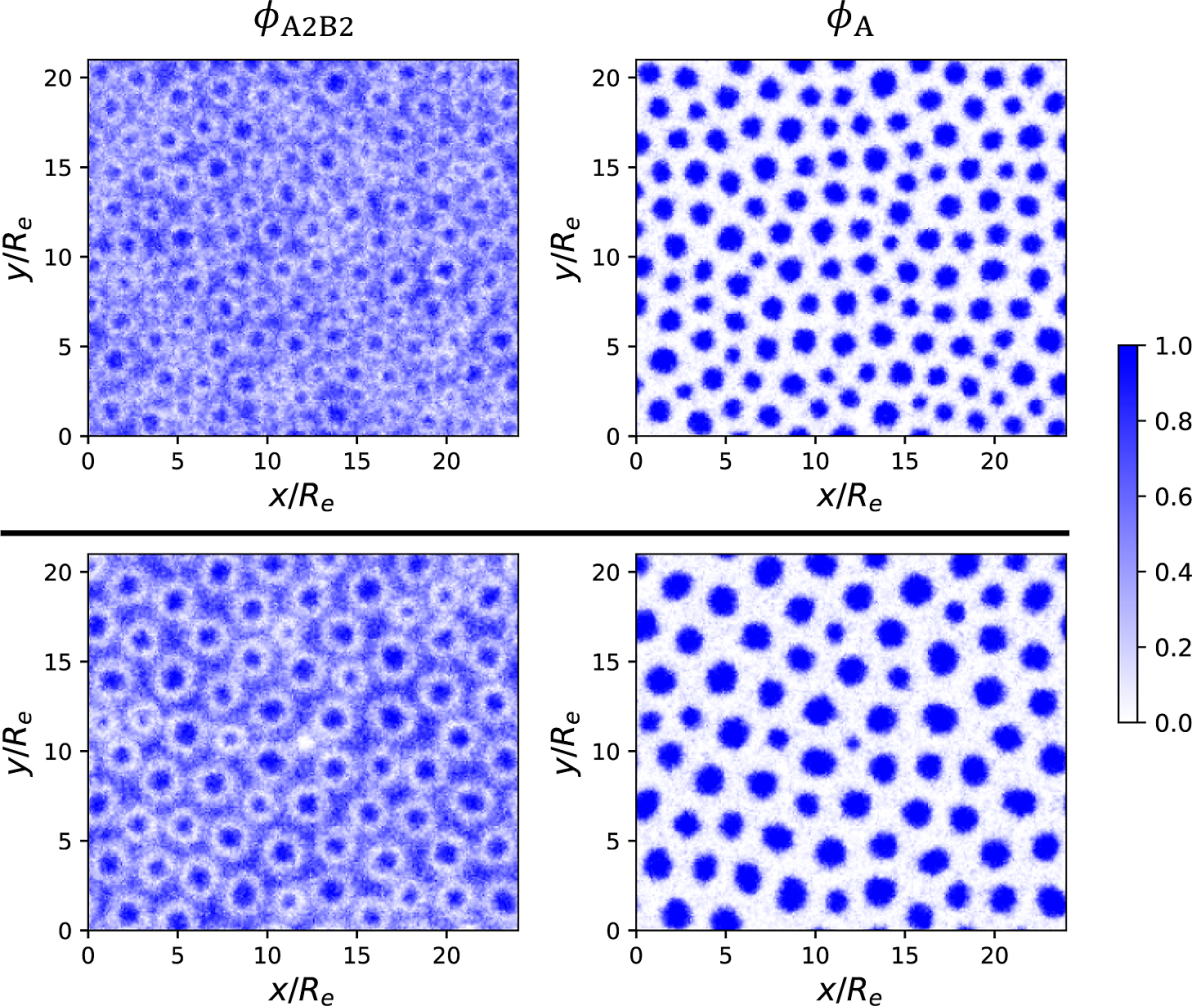
Final SCMF simulation snapshots at *t* = 40τ_*R*_ showing the density distributions of A_2_B_2_ copolymers (left column), and the total A blocks (right column), for the quenched system (top row) and the annealed system (bottom row). Both simulations target the state point—homogeneous HEX phase—indicated by the blue star in Figure 1c.

The outcome reflects the pronounced metastability of the single, homogeneous HEX structure throughout the entire range from the ODT to the final χ_AB_ *N*_1_ = 17.5 at ${\mathrm{\phi ̅}}_{2}=0.45$ . Owing to the diffusive dynamics of structure formation (and the finite k* at the spinodal predicted by RPA), the wavevector *k*_max_ of the fastest growing composition-fluctuation mode is finite along the annealing path. The resulting, incipient finite-*k* structure formed during annealing is itself metastable. The transient trapping of the system in this metastable structure hinders the progression toward macrophase separation, even though a significant part of the state trajectory during annealing falls within the two-phase coexistence region.

Extending our investigation to the case of *f*_2_ > *f*_1_, we perform quenching and annealing simulations targeting the two state points marked by the red ( ${\mathrm{\phi ̅}}_{2}=0.15$ , χ_AB_ *N*_1_ = 20) and blue ( ${\mathrm{\phi ̅}}_{2}=0.35$ , χ_AB_ *N*_1_ = 20) stars in Figure 3c, using processing conditions similar to those in the *f*_2_ = *f*_1_ case. The corresponding density snapshots are shown in Figures S6–S9, analogous to those in Figure 7, Figure 11, Figure 12, and Figure 13, respectively. Specific processing parameters are provided in the figure captions. For both quenched and annealed blends with *f*_2_ = 0.5, the final density profiles and their temporal evolution closely resemble those observed for *f*_2_ = 5/16. We assess the (meta)­stability of the single, homogeneous HEX phase using SCFT calculations, and present the results in a finely resolved phase diagram in Figure 10b, which is analogous to Figure 10a but constructed with the parameters used in Figure 3c. At ${\mathrm{\phi ̅}}_{2}=0.15$ , the HEX solution persists down to χ_AB_ *N*_1_ ≈ 12.6, which lies well above the spinodal value of χ_AB_ *N*_1_ ≈ 8.33; whereas at ${\mathrm{\phi ̅}}_{2}=0.35$ , the HEX solution remains accessible even below the spinodal of the DIS structure. The results from SCMF simulations and SCFT calculations support similar conclusions to those drawn for *f*_2_ = 0.3125. Therefore, we expect that the topographical feature of the free-energy landscape revealed here extends to other block-composition combinations. Notably, sparse local copolymer demixing is observed in the final density profiles of the annealed system at ${\mathrm{\phi ̅}}_{2}=0.35$ (Figure S9), despite the HEX phase being the equilibrium state at χ_AB_ *N*_1_ = 20 and remaining metastable down to below the spinodal point. Such demixing spots should originate from rare nucleation events, as they appear far less frequently than in the annealed system at ${\mathrm{\phi ̅}}_{2}=0.15$ .

The thermodynamic equilibrium within the wide two-phase coexistence region (DIS + HEX or HEX + HEX in Figure 10) consists of two macroscopic grains, with one exhibiting a large HEX phase and the other showing either a small HEX phase or the DIS phase, distinguished by their composition ${\mathrm{\phi ̅}}_{2}$ . The relative volume fractions of the coexisting phases are dictated by the lever rule. While small-scale demixing between the short and long copolymers can clearly be appreciated in the annealed systems shown in the bottom panels of Figures 7 and S6 the segregation remains local, leading to a final morphology at 40τ_*R*_ that deviates significantly from thermodynamic equilibrium. This raises the question of how processing conditions can be tailored to steer structure formation so that the system evolves toward equilibrium on a finite time scale.

To explore this, we focus on the phase diagram in Figure 10a and conduct two simulations at ${\mathrm{\phi ̅}}_{2}=0.25$ , quenching the system from the DIS phase to χ_AB_ *N*_1_ = 14.5 and 9.4469 at *t* = 0, marked as a red triangle and a red cross, respectively. These incompatibilities are chosen such that (i) the DIS structure is linearly unstable, and (ii) the homogeneous HEX structure is expected to be unstable at χ_AB_ *N*_1_ = 9.4469 but metastable at χ_AB_ *N*_1_ = 14.5. Both systems are evolved over an ultralong duration of 1000τ_*R*_.

Simulation snapshots of the system quenched to χ_AB_ *N*_1_ = 14.5 at representative times are shown in Figure 14. This state lies slightly above the triple line in Figure 10a, where a small and a large HEX phase coexist in equilibrium, and a homogeneous HEX structure is metastable. After quenching, the system quickly adopts a cylindrical morphology with well-mixed A_1_B_1_ and A_2_B_2_ copolymers. Even at *t* = 1000τ_*R*_, the A_1_B_1_ and A_2_B_2_ copolymers remain largely mixed, with local demixing emerging at a few irregular locations. In these small, locally demixed regions, A-rich cylinders dissolve due to the depletion of longer copolymers. At this state point, the equilibrium phase diagram predicts the formation of A_1_B_1_-rich, small cylindrical domains in the A_2_B_2_-depleted regions. However, only local disordered patches in the A-block density are observed in Figure 14 at *t* = 1000τ_*R*_. This discrepancy may arise because (i) the state point lies near the triple line, and fluctuations in the SCMF simulation are expected to shift the triple line toward slightly higher χ_AB_ *N*_1_, effectively placing the state point within the DIS + HEX region; and (ii) due to the limited size of the patches, the confined small HEX structure may experience geometric frustration, and these confinement effects can shift the phase-coexistence condition.

**Figure 14:**
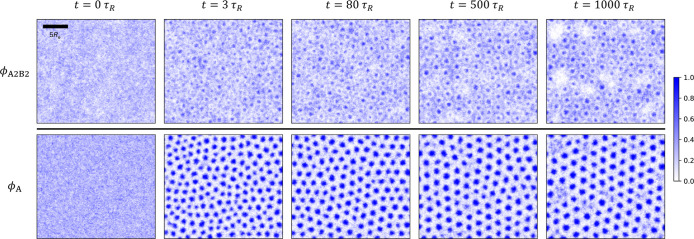
Quasi-2D SCMF simulation snapshots at representative time steps, showing the time evolution of A_2_B_2_-copolymer (top row), and total-A-block (bottom row) normalized density distributions, for the system quenched from the DIS phase to χ_AB_ *N*_1_ = 14.5 at ${\mathrm{\phi ̅}}_{2}=0.25$ in Figure 10a. Axis labels and ticks are the same as in Figure 7 and are omitted here. A scale bar is included in the graph at the top left to indicate the length scale 5*R*_e_.

To facilitate the analysis of length scales, we also compute structure factors based on the A density. The 2D structure factor, *S*(*k*_*x*_, *k*_*y*_), shown in Figure 15a displays sharp peaks with clear 6-fold symmetry, indicating a well-ordered homogeneous HEX pattern. The A-rich cylinders exhibit a relatively narrow size distribution and uniform intercolumnar spacing, as indicated by the sharp and narrow profile of *S*(*k*_∥_) in Figure 15a. Moreover, the peak positions, $k_{∥}=\frac{4\pi }{\sqrt{3}d_{c}}$ obtained by SCFT, for the small, large, and metastable, homogeneous HEX phases are indicated in the *S*(*k*_∥_) plot. The peak position of *S*(*k*_∥_) computed from the simulation agrees well with that for the SCFT-predicted metastable homogeneous HEX phase, and are sandwiched by those for the small and large HEX phases. These observations corroborates that the system is trapped in a metastable homogeneous HEX phase with a prolonged lifespan exceeding 1000τ_*R*_.

**Figure 15:**
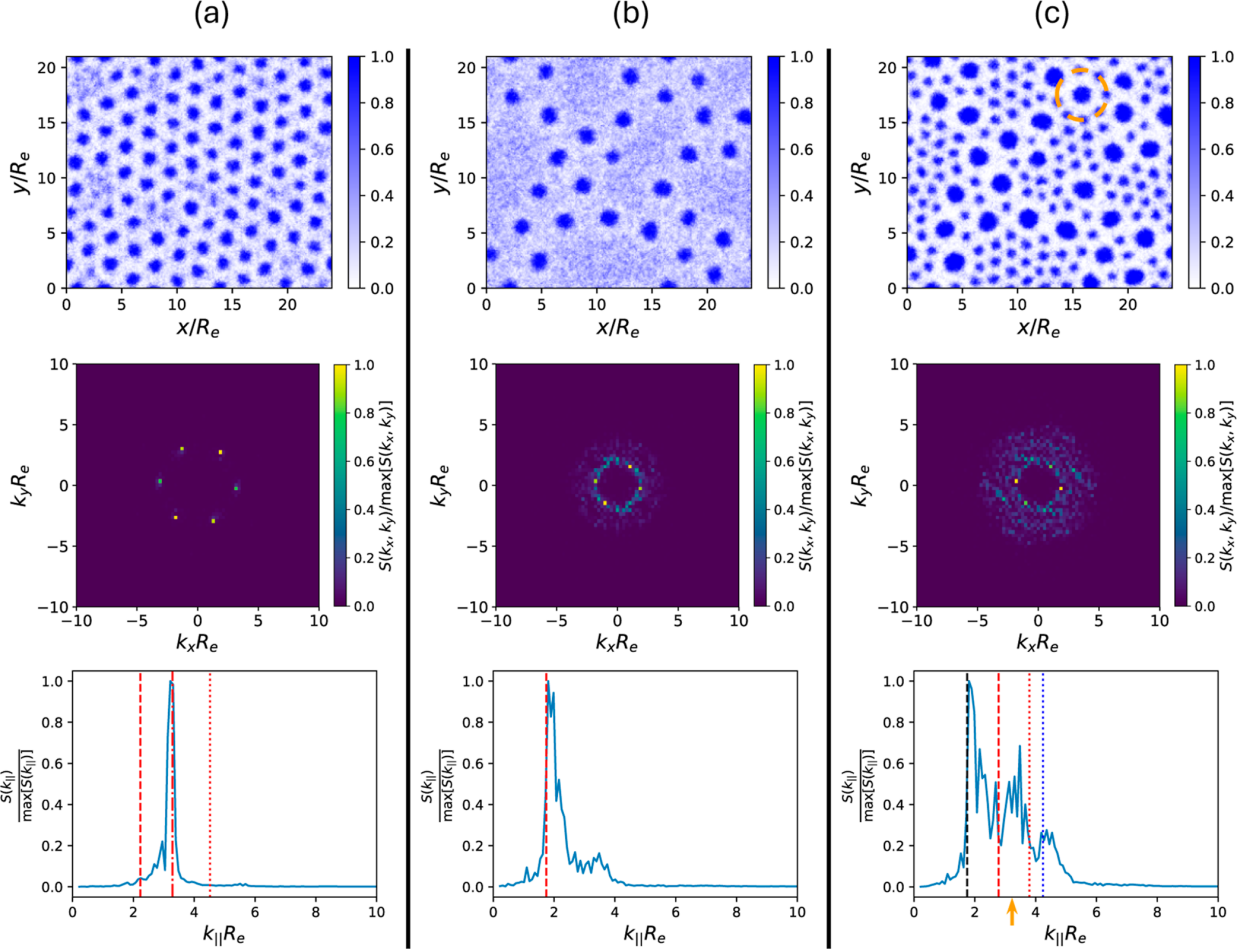
Normalized A-block density profiles (top), 2D structure factor *S*(*k*_*x*_, *k*_*y*_) (middle), and 1D structure factor *S*(*k*_∥_) (bottom). Panels (a,b) show the end results from simulations in Figure 14 (quench to χ_AB_ *N*_1_ = 14.5, ${\mathrm{\phi ̅}}_{2}=0.25$ ) and Figure 16 (quench to χ_AB_ *N*_1_ = 9.4469, ${\mathrm{\phi ̅}}_{2}=0.25$ ), respectively. Panel (c) shows the end results from a simulation quenched from the morphology in the middle panel to the state point, χ_AB_ = 17.5, ${\mathrm{\phi ̅}}_{2}=0.25$ , marked by the red star in Figure 10a, followed by evolution over 40τ_*R*_. The state points of all three panels lie within the two-phase coexistence region. In the *S*(*k*_∥_) plots shown in the bottom row, the vertical red dotted, dashed, and dash-dotted lines indicate the SCFT-predicted peak positions for the small, large, and metastable, homogeneous HEX phases, respectively. In panel (c), the black dashed line reproduces the red dashed line from panel (b), and the blue dotted line marks the SCFT-predicted peak position of the HEX phase at χ_AB_ *N*_1_ = 17.5, calculated using the ${\mathrm{\phi ̅}}_{2}$ value corresponding to the left boundary of the DIS + HEX region at χ_AB_ *N*_1_ = 9.4469. The orange arrow in the bottom panel of (c) corresponds to the peak position computed by assuming a *d*_*c*_ equal to the radius of the orange dashed circle in the top panel.

Representative simulation snapshots of the system quenched to χ_AB_ *N*_1_ = 9.4469 are presented in Figure 16. At this state point, the DIS phase and a large HEX phase coexist in equilibrium, whereas a homogeneous HEX phase is expected to be unstable. After quenching from the DIS structure, spatial inhomogeneity develops at a finite length scale (at *t* = 3τ_*R*_), forming A-rich cylinders. These cylinders coarsen over time (*t* = 80τ_*R*_) but do not exhibit clear HEX order (see Figure S10 for |ψ_6_|). Since the system is not trapped in a metastable homogeneous HEX phase, it approaches macrophase separation much more rapidly than in Figure 14. By *t* = 80τ_*R*_, copolymer demixing starts everywhere in the system, as expected for a spontaneous process. The domains continue to coarsen, leading to large A_1_B_1_-rich domains at *t* = 500τ_*R*_ and 1000τ_*R*_, indicative of clear macrophase separation.

**Figure 16:**
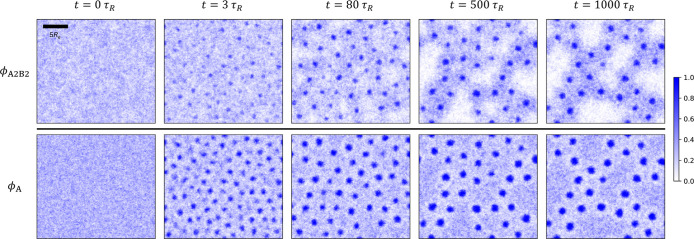
Quasi-2D SCMF simulation snapshots at representative time steps, showing the time evolution of A_2_B_2_-copolymer (top row), and total-A-block (bottom row) normalized density distributions, for the system quenched from the DIS phase to χ_AB_ *N*_1_ = 9.4469 at ${\mathrm{\phi ̅}}_{2}=0.25$ in Figure 10a. Axis labels and ticks are the same as in Figure 7 and are omitted here. A scale bar is included in the graph at the top left to indicate the length scale 5*R*_e_.

The corresponding structure factors of the A density at *t* = 1000τ_*R*_ are shown in Figure 15b. The dominant wavevector, related to the intercolumnar distance, *d*_*c*_, between A_2_B_2_-rich cylindrical domains via $k_{∥}=\frac{4\pi }{\sqrt{3}d_{c}}$ , appears as a bright ring in *S*(*k*_*x*_, *k*_*y*_) and as a sharp peak in *S*(*k*_∥_). Contributions from higher-frequency features exhibit much lower intensity, manifesting as a faint outer halo in *S*(*k*_*x*_, *k*_*y*_) and a shoulder in *S*(*k*_∥_). The peak position of *S*(*k*_∥_) closely matches that of the SCFT-predicted large HEX phase (red dashed line), albeit with a slight shift toward higher *k*_∥_, which is expected to vanish with further coarsening.

A quantitative measure of structural coarsening is provided by the average length scale

5 $$\mathrm{l̅}=\frac{2\pi }{\left\langle k_{∥}\right\rangle }$$

where ⟨*k*_∥_⟩ is defined in eq 4 with *S*(*k*_∥_) computed from the density of interest. To quantify the characteristic length scale of the A-block distribution, we evaluate *S*(*k*_∥_) and *l̅* from a binarized map defined by the threshold condition ϕ_A_(**r**) > 0.75ϕ_tot_(**r**), where ϕ_tot_(**r**) represents the total density. The threshold value of 0.75 is chosen to isolate A-rich cylindrical cores by excluding contributions from local, thermal composition fluctuations.

The temporal evolutions of *l̅* for the two ultralong simulations are shown in Figure 17a and c for the quench to χ_AB_ *N*_1_ = 14.5 and 9.4469, respectively. In both cases, *l̅* exhibits a clear increase over time, with a notably faster growth observed in Figure 17c, indicating more rapid coarsening of the average cylinder size. This trend aligns with the morphological evolution depicted in Figures 14 and 16. In Figure 17b,d, we present *l̅* computed based on a different binarization criterion [ϕ_A2_(**r**) + ϕ_B2_(**r**)]/ϕ_tot_(**r**) < 0.25, chosen to capture the length scale associated with spatial inhomogeneity in the copolymer distribution. This quantity grows slowly over time in Figure 17b, but more rapidly in Figure 17d, further highlighting the differing coarsening dynamics of the two systems as they progress toward the equilibrium two-phase coexistence. In particular, the length scale shown in Figure 17d is compatible with the growth law, $\mathrm{l̅}∼\sqrt{t}$ (red dashed fit), which characterizes the early stages of macrophase separation in two dimensions.^72,73^

**Figure 17:**
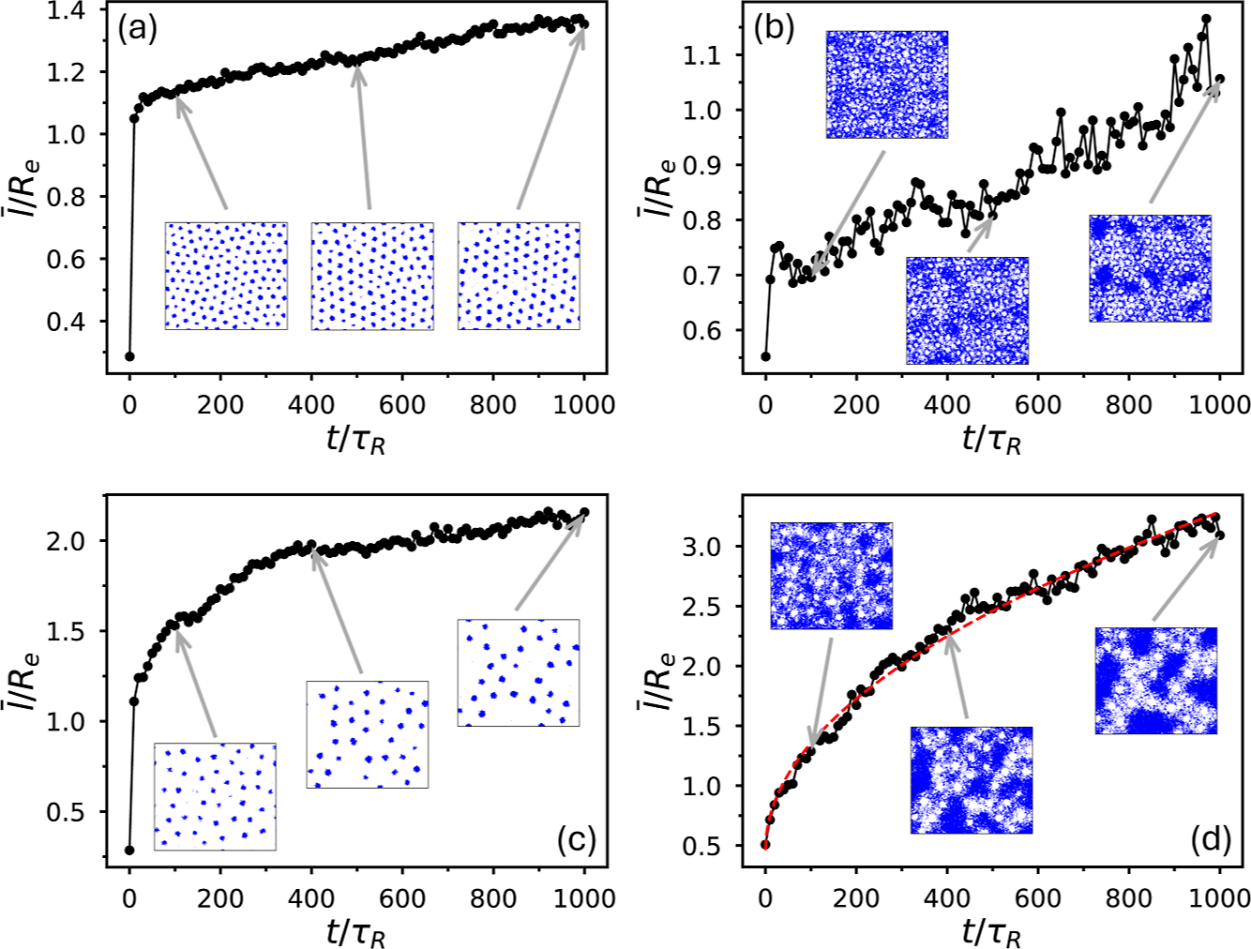
Average length scales, evaluated using eq 5, as a function of time for binarized maps defined by the conditions ϕ_A_(**r**) > 0.75ϕ_tot_(**r**) (microphase separation—a,c) and ϕ_A2_(**r**) + ϕ_B2_(**r**) < 0.25ϕ_tot_(**r**) (macrophase separation—b,d). Panels (a,b) show results from the simulation in Figure 14 (χ_AB_ *N*_1_ = 14.5, ${\mathrm{\phi ̅}}_{2}=0.25$ ), while panels (c,d) correspond to the simulation in Figure 16 (χ_AB_ *N*_1_ = 9.4469, ${\mathrm{\phi ̅}}_{2}=0.25$ ). Red dashed curve in (d) is fitted using the form $a\sqrt{t}+b$ .

Figure 15c also shows a morphology featuring macrophase-separated coexisting large and small cylinders, obtained by further quenching the morphology in Figure 15b to χ_AB_ *N*_1_ = 17.5 and allowing it to evolve for 40τ_*R*_. The formation of small cylinders introduces stronger high-*k*_∥_ contributions in the structure factors, resulting in a more pronounced and thicker ring in *S*(*k*_*x*_, *k*_*y*_) and a broader profile in *S*(*k*_∥_). In Figure 15c, red vertical lines with different styles indicate the SCFT-predicted peak positions for the coexisting, small and large HEX phases. The *S*(*k*_∥_) of the simulated, macrophase-separated morphology exhibits peaks that extend beyond the range enclosed by the red dotted and dashed lines on both the low- and high-*k*_∥_ sides, indicating a pronounced length-scale disparity relative to the equilibrium morphology at χ_AB_ *N*_1_ = 17.5. This mismatch arises because the copolymer concentrations in the two macrophase-separated phases (A_2_B_2_-poor and A_2_B_2_-rich) were initially established at χ_AB_ *N*_1_ = 9.4469, where the ${\mathrm{\phi ̅}}_{2}$ contrast between coexisting phases is significantly greater than at χ_AB_ *N*_1_ = 17.5 (see Figure 10a). After quenching to χ_AB_ *N*_1_ = 17.5, the polymer chains require substantially more than 40τ_*R*_ to redistribute and reach the new equilibrium concentrations. As a result, the copolymer concentrations in Figure 15c remain close to their previous coexistence values at χ_AB_ *N*_1_ = 9.4469. For clear comparison, the red dashed line from *S*(*k*_∥_) in Figure 15b is reproduced as a black dashed line in Figure 15c. The alignment between the dominant peak position and the black dashed line indicates that the average *d*_*c*_ between large cylinders is nearly identical in the two morphologies in Figure 15b and c. The blue dashed line denotes the SCFT-predicted peak position of the HEX morphology at χ_AB_ *N*_1_ = 17.5 and ${\mathrm{\phi ̅}}_{2}$ = 0.0281. This ${\mathrm{\phi ̅}}_{2}$ value corresponds to the left boundary of the DIS + HEX region at χ_AB_ *N*_1_ = 9.4469. As anticipated, the high-*k*_∥_ peaks—associated with the *d*_*c*_ between small cylinders—approximately align with this blue dashed line.

The peaks centered around *k*_∥_ *R*_e_ ≈ 0.32 correspond to intermediate length scales, such as the *d*_*c*_ between large and small cylinders at grain boundaries. This spacing is illustrated by the radius of the orange dashed circle in the top panel of Figure 15c, providing an estimate of the *d*_*c*_ between large and small cylinders. Using this radius as *d*_*c*_, the corresponding peak position—indicated by an orange arrow in the bottom panel—aligns well with the observed intermediate peaks.

Compared to the bottom panel of Figure 9 (the annealed system), the morphology in Figure 15c features aggregates of large or small cylinders, forming significantly larger grains. Although the cylinder sizes in the small and large HEX grains have not yet reached their equilibrium values, this represents a macrophase-separated state closer to thermodynamic equilibrium. In particular, the morphology shown in Figure 15c resembles that observed in TEM images of macrophase-separated binary blends of sphere-forming diblock copolymers reported in an early experimental study.^49^ At true thermodynamic equilibrium with complete macrophase separation, *S*(*k*_∥_) is expected to exhibit a more pronounced bimodal feature, with peaks closer to the red dotted and dashed lines. However, further coarsening from the morphology in Figure 15c to reach true equilibrium requires (i) all A_2_B_2_-rich cylinders to aggregate into a single, well-ordered HEX grain, and (ii) the cylinders in the small and large HEX phases to adjust their sizes to the equilibrium values through chain exchange. Due to the stronger segregation, the typical time scale of such processes would be much longer than the 1000τ_*R*_ required to observe partial macrophase separation between DIS and HEX at χ_AB_ *N*_1_ = 9.4469, as shown in Figure 15b. Consequently, achieving true thermodynamic equilibrium for macroscopic HEX + HEX phase coexistence is challenging, even on experimentally accessible time scales.

## Conclusion

In this work, we have investigated process-dependent structure formation in binary A_1_B_1_/A_2_B_2_ diblock copolymer blends using SCFT and particle-based simulations. Our study focuses on how processing pathways, coupled with micro- and macrophase separation, govern the resulting (meta)­stable structures. Using SCFT and RPA, we first construct equilibrium phase diagrams, identify the parameter region exhibiting macrophase separation between small- and large-period HEX phases, and determine the stability limits (spinodals) of the DIS and single, homogeneous HEX phases. We then employ SCMF simulations to examine the kinetics of structure formation under two processing protocols: rapid quenching and gradual thermal annealing.

Our quasi-2D SCMF simulations show that quenching blends from the DIS phase into the HEX + HEX coexistence region traps the system in a long-lived metastable single, homogeneous HEX phase with narrowly distributed cylinder sizes. In contrast, annealing to the same thermodynamic state point results in local demixing and partial HEX + HEX macrophase separation. Due to this local demixing during annealing, the final cylindrical morphology exhibits a clear bimodal feature with two distinct size groups, which consequently leads to weaker hexagonal order. Our analysis further reveals that this process-dependent behavior arises from the (near) loss of metastability of the single, homogeneous HEX phase in the DIS + HEX coexistence region and the concomitant tendency toward macrophase separation during annealing. Qualitatively consistent behaviors are observed when a short cylinder-forming copolymer is mixed with a long copolymer that is either cylinder- or lamella-forming, suggesting that the uncovered topography of the free-energy landscape holds across a broad range of the thermodynamic states with HEX + HEX phase coexistence.

Although this work focuses on 1D and 2D ordered phases, we expect the topographical features of the free-energy landscape—and hence the process-dependent nonequilibrium behavior—to extend to other morphologies and more complex multicomponent formulations. This work illustrates that the structures obtained by processing and their dependence on the thermodynamic parameters of the final state may significantly differ from the equilibrium phase behavior.^56,74^ Nevertheless, the SCFT free-energy functional not only offers a straightforward framework for constructing equilibrium phase diagrams of copolymer systems, but also provides topographical information about the free-energy landscape, which is essential for understanding the metastability of spatially modulated structures^75^ and the transformation pathways connecting them.

Our study provides a deeper understanding of structure–processing–property relationships in copolymer blends and offers insights into process-directed self-assembly of copolymer-based materials for advanced applications. In a follow-up study – Paper II^64^ – we examine how binary copolymer blends can be used to control the pore size of integral-asymmetric, isoporous block copolymer membranes via self-assembly by nonsolvent-induced phase separation (SNIPS).

## Supplementary Material



## Footnotes

a In the context of two coexisting phases, a *X*_*i*_-rich phase (*X*_*i*_ = A_1_B_1_ or A_2_B_2_) is defined as the one with a higher *X*_*i*_ content relative to the other coexisting phase. By this convention, in an A_1_B_1_-rich phase, we refer to the A_1_B_1_ copolymer as the rich copolymer species, even if it may have a lower absolute volume fraction than the A_2_B_2_ copolymer in the same phase. In contrast, in the context of microdomains, a *C*_*i*_-rich domain is defined as one where *ϕ*_*C*_*i*__(**r**)>*ϕ*_non‑*C*_*i*__(**r**), with *C*_*i*_ denoting A_1_, B_1_, A_2_, B_2_, or any combination thereof.

b We note that even if the homogeneous structure is linearly unstable with respect to macrophase separation, *k** = 0, the fastest growing mode will occur at a finite wavevector because of the diffusive dynamics.

c Compared to the microphase-separated morphology obtained by quenching in Figure 7, the corresponding structure in Figure 13 exhibits greater disparity in cylinder sizes and a less ordered arrangement. This phenomenon is rooted in an increase of segregation degree induced by the larger concentration of the longer A_2_B_2_ copolymer—a point that is also addressed in paper II.^64^

## Notes

Self-Consistent Field Theory (SCFT). Random-Phase Approximation (RPA). Visualization of System Evolution and Free-Energy Landscapes. Additional Figures (PDF)
