# Supplementary material for: Process-Directed Self-Assembly of Copolymer Blends: I. Micro- and Macrophase Separation
Source: Macromolecules. 2025 Oct 24;58(21):11523–38. doi: 10.1021/acs.macromol.5c02090 (PMC12613812; doi:10.1021/acs.macromol.5c02090)
Supplement: Supplementary file 1 [file ma5c02090_si_001.pdf]

Supporting Information

for

Process-directed self-assembly of copolymer blends: I. Micro- and macrophase separation

Jiayu Xie* and Marcus Müller*

Institute for Theoretical Physics, Georg-August University of Göttingen, 37077 Göttingen, Germany

E-mail: jiayu.xie@theorie.physik.uni-goettingen.de; mmueller@theorie.physik.uni-goettingen.de

This PDF includes:

- Self-Consistent Field Theory (SCFT)
- Random-Phase Approximation (RPA)
- Visualization of System Evolution and Free-Energy Landscapes
- Additional Figures

Self-Consistent Field Theory (SCFT)

We consider an incompressible binary mixture of n_1 short diblock copolymers (A_1B_1) and n_2 long diblock copolymers (A_2B_2) in a volume V. We model the polymers as discrete Gaussian chains[1] composed of coarse-grained beads connected by harmonic bonds. Each A_1B_1 chain consists of N_{A1} A beads and N_{B1} B beads, giving a total of $N_1 = N_{A1} + N_{B1}$ beads per chain. Similarly, each A_2B_2 chain comprises N_{A2} A beads and N_{B2} B beads, with $N_2 = N_{A2} + N_{B2}$ in total. The block compositions (volume fractions) are given by $f_1 = f_{A1} = N_{A1}/N_1$ and $f_2 = f_{A2} = N_{A2}/N_2$. For convenience, a reference chain length N_0 can be defined, and the length ratios between different chains to the reference can subsequently be defined as $\gamma_1 = N_1/N_0$ and $\gamma_2 = N_2/N_0$. Throughout this work, we set the length of the short A_1B_1 diblock copolymer as the reference $N_0 = N_1$ (hence $\gamma_1 = 1$), and $N_1 = 64$ is kept fixed for all theoretical calculations and particle-based simulations.

In self-consistent field-theory (SCFT) and random-phase approximation (RPA) calculations, the system is assumed to have a uniform segment density ρ_0. The average concentrations of the two copolymers are given by $\bar{\phi}_1 = n_1 N_1/\rho_0 V$ and $\bar{\phi}_2 = n_2\gamma_2 N_1/\rho_0 V$, where $\rho_0 V = n_1 N_1 + n_2 \gamma_2 N_1$. For simplicity, all bonds in the system are assumed to have the same statistical segment length, b. The bonded potential for a single bond connecting segments t and $t+1$ is:

$$\mathcal{H}_{\mathrm{bond}} = \frac{3k_{\mathrm{B}}T}{2b^2}|\mathbf{r}_{t+1} - \mathbf{r}_t|^2 \,. \tag{S1}$$

The unit of length of the system is chosen as the root-mean-squared end-to-end distance $R_e = b\sqrt{N_1 - 1}$ of a noninteracting linear reference chain with N_1 beads and $N_1 - 1$ bonds.

We first formulate SCFT in the canonical ensemble, where the average concentrations, $\bar{\phi}_1$ and $\bar{\phi}_2$, are the thermodynamic control parameters. The Helmholtz free energy density can be written as:[2,3]

$$
\begin{aligned}
\frac{N_1\mathcal{F}}{\rho_0 V k_{\mathrm{B}}T} =& -\bar{\phi}_1 \ln\frac{Q_1}{\bar{\phi}_1} - \frac{\bar{\phi}_2}{\gamma_2}\ln\frac{Q_2}{\bar{\phi}_2} - \frac{1}{V}\int \mathrm{d}\mathbf{r}\ \left[N_1\omega_{\mathrm{A}}(\mathbf{r})\phi_{\mathrm{A}}(\mathbf{r}) + N_1\omega_{\mathrm{B}}(\mathbf{r})\phi_{\mathrm{B}}(\mathbf{r})\right. \\
&\left. -\chi_{\mathrm{AB}}N_1\phi_{\mathrm{A}}(\mathbf{r})\phi_{\mathrm{B}}(\mathbf{r}) + \eta(\mathbf{r})N_1\left(1-\phi_{\mathrm{A}}(\mathbf{r})-\phi_{\mathrm{B}}(\mathbf{r})\right)\right],
\end{aligned}
\tag{S2}
$$

where VQ_i is the single-chain partition function of copolymer i ($i = 1$ or 2), including its translational entropy; $\phi_\alpha(\mathbf{r})$ is the density field of segment species $\alpha \in \{\mathrm{A},\mathrm{B}\}$; $\omega_\alpha(\mathbf{r})$ is the auxiliary field conjugate to $\phi_\alpha(\mathbf{r})$; $\chi_{\alpha\beta}$ is the Flory-Huggins interaction parameter; and $\eta(\mathbf{r})$ is the pressure-like Lagrange field enforcing incompressibility.

Extremizing Equation S2 leads to the following SCFT equations:

$$\omega_{\mathrm{A}}(\mathbf{r}) = \chi_{\mathrm{AB}}\phi_{\mathrm{B}}(\mathbf{r}) + \eta(\mathbf{r}), \tag{S3}$$

$$\omega_{\mathrm{B}}(\mathbf{r}) = \chi_{\mathrm{AB}}\phi_{\mathrm{A}}(\mathbf{r}) + \eta(\mathbf{r}), \tag{S4}$$

$$\phi_{\mathrm{A}}(\mathbf{r}) = \frac{\bar{\phi}_1}{Q_1N_1}\sum_{t=1}^{N_{\mathrm{A}1}} q_1(t,\mathbf{r})e^{\omega_{\mathrm{A}}(\mathbf{r})}q_1^\dagger(t,\mathbf{r}) + \frac{\bar{\phi}_2}{Q_2N_2}\sum_{t=1}^{N_{\mathrm{A}2}} q_2(t,\mathbf{r})e^{\omega_{\mathrm{A}}(\mathbf{r})}q_2^\dagger(t,\mathbf{r}), \tag{S5}$$

$$\phi_{\mathrm{B}}(\mathbf{r}) = \frac{\bar{\phi}_1}{Q_1N_1}\sum_{t=N_{\mathrm{A}1}+1}^{N_1} q_1(t,\mathbf{r})e^{\omega_{\mathrm{B}}(\mathbf{r})}q_1^\dagger(t,\mathbf{r}) + \frac{\bar{\phi}_2}{Q_2N_2}\sum_{i=N_{\mathrm{A}2}+1}^{N_2} q_2(t,\mathbf{r})e^{\omega_{\mathrm{B}}(\mathbf{r})}q_2^\dagger(t,\mathbf{r}), \tag{S6}$$

$$\phi_{\mathrm{A}}(\mathbf{r}) + \phi_{\mathrm{B}}(\mathbf{r}) = 1. \tag{S7}$$

where $\alpha_i(t)$ specifies the segment type of the tth segment on molecule species i.

In Equation S5 and Equation S6, $q_i(t,\mathbf{r})$ represents the chain propagator for copolymer i, which is obtained by solving the following iterative equation:

$$q_i(t+1,\mathbf{r}_{t+1}) = e^{-\omega_{\alpha_i(t+1)}(\mathbf{r}_{t+1})}\int \mathrm{d}\mathbf{r}_t\ p_{t+1,t}(\mathbf{r}_{t+1}-\mathbf{r}_t)q_i(t,\mathbf{r}_t), \tag{S8}$$

with the initial condition $q_i(1,\mathbf{r}) = e^{-\omega_{\alpha_i(1)}(\mathbf{r})}$ where $\alpha_i(1)$ denotes the type of segment $t = 1$

on copolymer i. The bond transition probability from the tth to $(t+1)^{\mathrm{th}}$ beads is written as:

$$p_{t+1,t}(\mathbf{r}_{t+1}-\mathbf{r}_t)=\left(\frac{3}{2\pi b^2}\right)^{\frac{3}{2}}\exp\left(-\frac{3|\mathbf{r}_{t+1}-\mathbf{r}_t|^2}{2b^2}\right). \tag{S9}$$

The function $q_i^\dagger(t,\mathbf{r})$ in Equation S5 and Equation S6 is the adjoint propagator to $q_i(t,\mathbf{r})$, which is obtained by iterating Equation S8 in the opposite direction along the chain.

The single-molecule partition function of copolymer i is given by

$$\mathcal{Q}_i \;=\; \frac{1}{V}\int \mathrm{d}\mathbf{r}\; q_i(t,\mathbf{r})e^{\omega_{\alpha_i(t)}(\mathbf{r})}q_i^\dagger(t,\mathbf{r}) \tag{S10}$$

for arbitrary t.

To determine phase coexistence, it is convenient to work in the semigrand canonical ensemble, where the thermodynamic control parameters are the chemical potentials of the two copolymers μ_1 and μ_2. The semigrand potential density $\mathcal{G}$ takes the form:[2,3]

$$\begin{aligned}\frac{N_1\mathcal{G}}{\rho_0 V k_\mathrm{B}T} = &-e^{\mu_1/k_\mathrm{B}T}Q_1 - e^{\mu_2/k_\mathrm{B}T}Q_2 - \frac{1}{V}\int \mathrm{d}\mathbf{r}\;\left[N_1\omega_\mathrm{A}(\mathbf{r})\phi_\mathrm{A}(\mathbf{r}) + N_1\omega_\mathrm{B}(\mathbf{r})\phi_\mathrm{B}(\mathbf{r})\right.\\ &\left.-\chi_\mathrm{AB}N_1\phi_\mathrm{A}(\mathbf{r})\phi_\mathrm{B}(\mathbf{r}) + \eta(\mathbf{r})N_1\left(1-\phi_\mathrm{A}(\mathbf{r})-\phi_\mathrm{B}(\mathbf{r})\right)\right],\end{aligned} \tag{S11}$$

Similar to the average concentrations, only one of the two chemical potentials is independent due to the incompressibility constraint, which allows us to set $\mu_1 = 0$. Extremizing Equation S11, we obtain the same set of SCFT equations as in Eqs. S3-S7, except that the equations calculating the density fields (Equation S5 and Equation S6) are modified to:

$$\phi_\mathrm{A}(\mathbf{r}) = \frac{1}{N_1}\sum_{t=1}^{N_{\mathrm{A}1}} q_1(t,\mathbf{r})e^{\omega_\mathrm{A}(\mathbf{r})}q_1^\dagger(t,\mathbf{r}) + \frac{e^{\mu_2/k_\mathrm{B}T}}{N_1}\sum_{t=1}^{N_{\mathrm{A}2}} q_2(t,\mathbf{r})e^{\omega_\mathrm{A}(\mathbf{r})}q_2^\dagger(t,\mathbf{r}), \tag{S12}$$

$$\phi_\mathrm{B}(\mathbf{r}) = \frac{1}{N_1}\sum_{t=N_{\mathrm{A}1}+1}^{N_1} q_1(t,\mathbf{r})e^{\omega_\mathrm{B}(\mathbf{r})}q_1^\dagger(t,\mathbf{r}) + \frac{e^{\mu_2/k_\mathrm{B}T}}{N_1}\sum_{t=N_{\mathrm{A}2}+1}^{N_2} q_2(t,\mathbf{r})e^{\omega_\mathrm{B}(\mathbf{r})}q_2^\dagger(t,\mathbf{r}). \tag{S13}$$

After solving the SCFT equations, the average concentrations are given by $\bar{\phi}_1 = Q_1$ and $\bar{\phi}_2 = 1-\bar{\phi}_1$.

To construct equilibrium phase diagrams, we numerically solve Eqs. S3, S4, S7, S12 and S13 for a set of candidate phases and compare their semigrand potentials. During the optimization of the field variables, the dimensions of the computational box are optimized concurrently using gradient descent followed by variable-cell Anderson mixing to accelerate convergence.[4,5] The convergence criteria are: $\max_{\mathbf{r}} |1 - \phi_{\mathrm{A}}(\mathbf{r}) - \phi_{\mathrm{B}}(\mathbf{r})| < 10^{-5}$, err $< 10^{-5}$, where err denotes the relative L2-norm error of the auxiliary fields,[5] and the change in free energy or semigrand potential density relative to the previous iteration is less than 10^{-6}. The maximum number of iterations is set to 2000; if the criteria are not satisfied within this limit, the calculation is deemed unconverged.

In this work, we restrict our attention to ordered structures with density variations confined to one-dimensional (1D) and two-dimensional (2D). Accordingly, the candidate phases considered are lamellae (LAM), hexagonally packed cylinders (HEX), and the disordered (DIS) phase.

Random-Phase Approximation (RPA)

The spinodal point, at which the disordered state becomes linearly unstable, provides useful information for studying the ordering process of a system. For polymer blends, the spinodal can be determined using the RPA.[6–9] The major task in RPA is to evaluate the inverse collective structure factor, $\mathbb{S}^{-1}(\mathbf{k})$, which corresponds to the second-order coefficient matrix of the expansion of the free energy density (Equation S2) with respect to the composition fluctuations, $\delta\phi_\alpha(\mathbf{k})$, with wavevector k:

$$\frac{\mathcal{F}[\phi]-\mathcal{F}_{\mathrm{homo}}}{k_{\mathrm{B}}T\rho_0 V}=\frac{1}{2}\sum_{\mathbf{k}}\sum_{\alpha,\beta}\delta\phi_\alpha(\mathbf{k})\mathbb{S}^{-1}_{\alpha\beta}(\mathbf{k})\delta\phi_\beta(-\mathbf{k})+\mathcal{O}\left(\delta\phi_\alpha^3\right), \tag{S14}$$

where $\mathcal{F}_{\mathrm{homo}}$ denotes the free energy for the homogeneous phase. For the binary blends with two chemically distinct components, i.e., A and B, there is only one independent composition fluctuation due to the incompressibility constraint. As a result, the inverse collective structure factor reduces to a scalar, $S^{-1}(\mathbf{k})$. Following the procedure of RPA for polymer mixtures,[9] the $S^{-1}(\mathbf{k})$ for binary A_1B_1/A_2B_2 blends is written as

$$S^{-1}(\mathbf{k})=\frac{U}{D}-2\chi_{\mathrm{AB}} \tag{S15}$$

where

$$U=N_1[g_{\mathrm{A1A1}}(k)+2g_{\mathrm{A1B1}}(k)+g_{\mathrm{B1B1}}(k)]\bar{\phi}_1+N_2[g_{\mathrm{A2A2}}(k)+2g_{\mathrm{A2B2}}(k)+g_{\mathrm{B2B2}}(k)]\bar{\phi}_2 \tag{S16}$$

and

$$\begin{aligned}D=&N_1^2[g_{\mathrm{A1A1}}(k)g_{\mathrm{B1B1}}(k)-g^2_{\mathrm{A1B1}}(k)]\bar{\phi}_1^2+N_2^2[g_{\mathrm{A2A2}}(k)g_{\mathrm{B2B2}}(k)-g^2_{\mathrm{A2B2}}(k)]\bar{\phi}_2^2\\&+N_1N_2[g_{\mathrm{A1A1}}(k)g_{\mathrm{B2B2}}(k)+g_{\mathrm{A2A2}}(k)g_{\mathrm{B1B1}}(k)-2g_{\mathrm{A1B1}}(k)g_{\mathrm{A2B2}}(k)]\bar{\phi}_1\bar{\phi}_2\end{aligned} \tag{S17}$$

For fluctuations around the spatially homogeneous, disordered phase, the functions $g_{\alpha\beta}$ depend only on the magnitude of the wavevector (k), and are related to correlation functions of ideal, noninteracting discrete Gaussian chains:

$$g_{\alpha\alpha}(k) = \frac{2p(k)[p^{N_\alpha}(k) - 1] - N_\alpha p^2(k) + N_\alpha}{N_i^2[p(k) - 1]^2}, \tag{S18}$$

and

$$g_{\alpha\beta}(k) = \frac{p(k)\left[p^{N_\alpha}(k) - 1\right]\left[p^{N_\beta}(k) - 1\right]}{N_i^2[p(k) - 1]^2}. \tag{S19}$$

Here, N_i denotes the total length of the polymer chain containing the block described by the correlation function, and $p(k)$ is the Fourier transform of the bond transition probability, Equation S9, $p(k) = \exp\left(-\frac{k^2 b^2}{6}\right)$.

The stability limit of the disordered phase is identified at the point where the minimum of S^{-1} equals zero at a wavevector k^*. A nonzero k^* indicates an instability against a composition fluctuation mode with a finite, microscopic length scale, whereas $k^* = 0$ corresponds to a mode with a diverging, macroscopic length scale.

Near $k = 0$, $S^{-1}(\mathbf{k})$ exhibits oscillatory behavior, which complicates the precise determination of the transition point where k^* changes from nonzero to zero. To address this, we impose a cutoff of $k^* R_e \leq 0.05$, treating all values below this threshold as indicative of a macroscopic mode.

Visualization of System Evolution and Free-Energy Landscapes

To provide an intuitive physical picture, Figure S2 summarizes a simplified visual explanation of the process dependence in structure formation observed at the state points marked by the red stars in Figure 10(a) and (b). In Figure S2, the free-energy landscape is represented by a 1D curve, and the system morphology/configuration is denoted as a red circle. Within the same row, the morphology and the free-energy landscape evolve from the leftmost to the rightmost column. The finely-resolved phase diagrams in Figure 10 are reproduced in Figure S1 with additional state points marked by different circled numbers. The symbol under each small penal in Figure S2 indicates the state point on the phase diagrams that the depicted free-energy landscape describes.

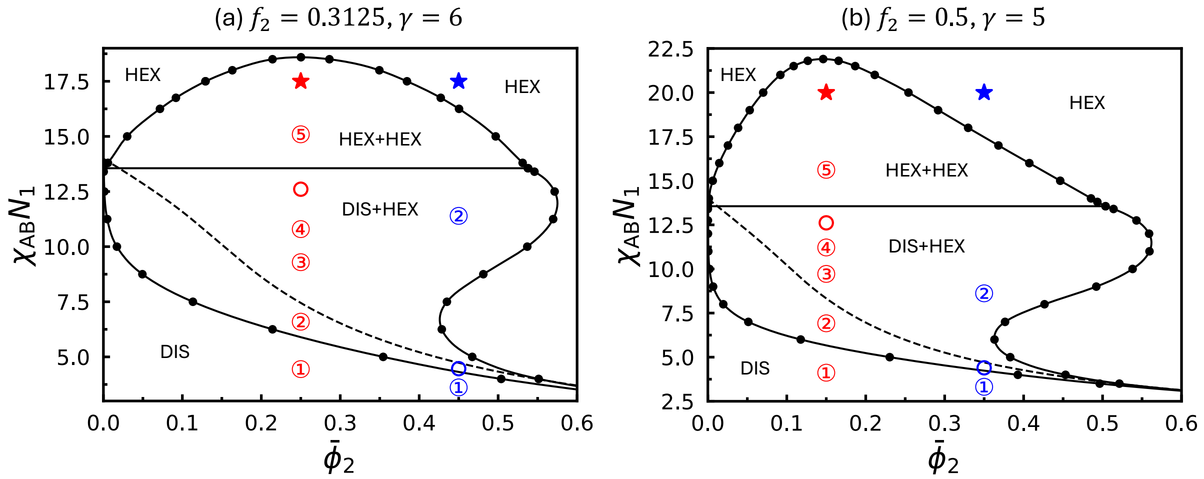

Figure S1: Finely resolved phase diagrams reproduced from Figure 10, with additional symbols marking representative state points that are referenced in Figure S2.

Figure S2(a) illustrates the evolution induced by quenching the blend from state points below the order-disorder-transition (ODT), e.g. the red ①, to the red star in Figure S1(a) or (b). At the red ①, the global minimum of the free energy is the DIS phase. After the abrupt change in free-energy topography induced by quenching, the HEX+HEX 2ϕ state emerges as the global minimum of the free energy, whereas the single HEX phase appears as a local

minimum. The spatial modulation of density in the single HEX phase has a significantly smaller characteristic length scale compared to the macrophase-separated state. Therefore, as the system morphology gradually evolves toward the low-free-energy states via coarsening, it first approaches the local minimum of the free energy corresponding to the single HEX phase and becomes trapped in its valley. Subsequent progression toward the global minimum (2ϕ) requires overcoming free-energy barriers associated with migrating the two copolymers across a macroscopic length scale in a spatially modulated morphology, which is a nucleation process and occurs only over very long times.

In contrast, annealing leads to a qualitatively different topographical evolution of the free energy, as depicted in Figure S2(b). During annealing, the system traverses different state points as represented by the red ②, ③ and ④, before reaching the target point (red star). At ②, which is located between the ODT and the spinodal of the DIS phase, the DIS+HEX 2ϕ state is the global minimum while the DIS phase maintains its metastability. When $\chi_{\mathrm{AB}}N_1$ passes the DIS spinodal (e.g., at red ③), the DIS phase becomes unstable and spontaneous phase separation takes place initially at a finite length scale. At this stage, the single HEX phase is not metastable and therefore the system slowly progresses toward macrophase separation through local demixing (e.g., at red ④). When the single HEX phase emerges as a local minimum as $\chi_{\mathrm{AB}}N_1$ further passes the HEX spinodal (red circle in Figure S1(a) or (b)), the system morphology has reached a point outside of its free-energy valley, resulting in a partially demixed morphology composed of large and small cylinders. Figure S2(a) and (b) explain the process-dependent morphological evolution observed in Figures 11, 12, S7 and S8.

Different from annealing the blends from the DIS phase to the red stars in Figure S1, annealing them to the blue stars primarily lead to microphase separation without substantial local demixing of the two copolymers. This is explained in Figure S2(c): the single HEX phase has already become a local free-energy minimum at state points between the ODT and the DIS spinodal. Hence, as the DIS phase loses its metastability, the system can evolve

to and be trapped at the metastable, single HEX phase.

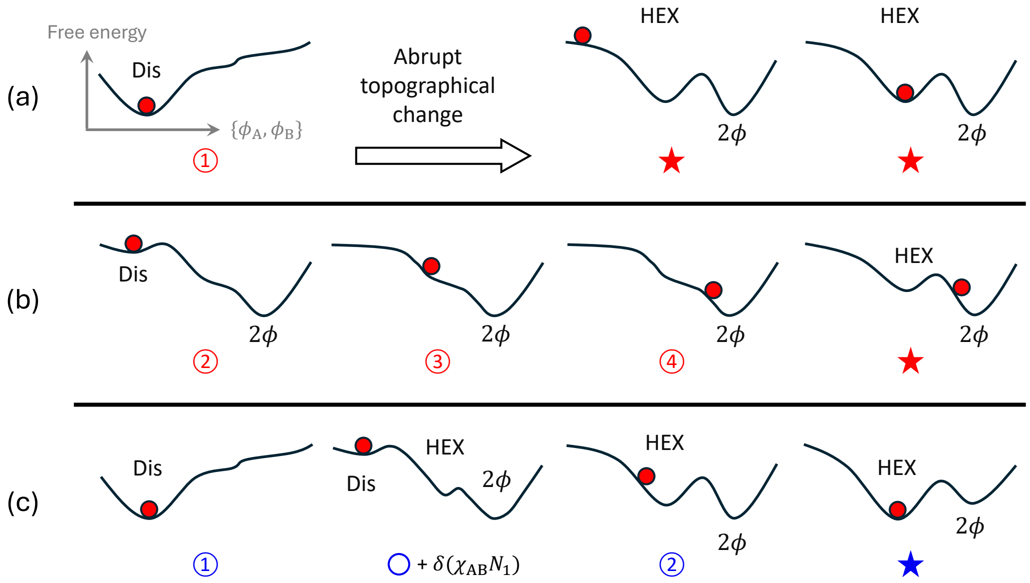

Figure S2: Visual illustration of the time evolution of the free-energy landscape and system morphology (left to right) under different processing conditions and/or along different phase paths: (a) quenching from the DIS phase to the red stars, (b) annealing from the DIS phase to the red stars, and (c) annealing from the DIS phase to the blue stars in Figure S1. The free-energy landscape is schematically represented by a 1D curve, and the system morphology/configuration is denoted as a red circle. The symbol under each small penal indicates the state point on the phase diagrams that the depicted free-energy landscape describes. In (c), $\bigcirc + \delta(\chi_{AB}N_1)$ represents a point located just above the blue circle but remaining below the spinodal of the disordered phase.

We note that the schematics of free-energy landscape in Figure S2 are drastically simplified by (1) representing a high-dimensional free energy functional in 1D and (2) omitting small-scale free-energy barriers associated with individual fusion events. Nevertheless, it conveys the essential picture that is required to rationalize the process-dependent morphological behavior.

Additional Figures

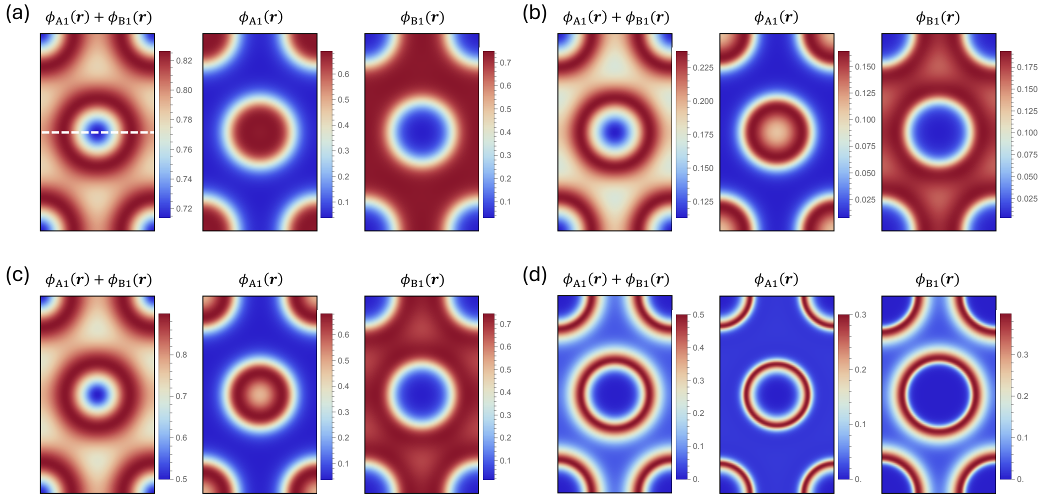

Figure S3: 2D densities for the A_1B_1 copolymers, A_1 blocks and B_1 blocks in the HEX phases formed in the binary A_1B_1/A_2B_2 blends with $f_1 = f_2 = 0.3125$, $\chi_{AB}N_1 = 17.5$, and $\{\gamma_2, \bar{\phi}_2\}$=(a)$\{1.5, 0.2\}$, (b)$\{1.5, 0.8\}$, (c)$\{6, 0.2\}$ and (d)$\{6, 0.8\}$. The white dashed line in (a) illustrates the path in the HEX unit cell, along which the 1D curves in Figure S4 are extracted.

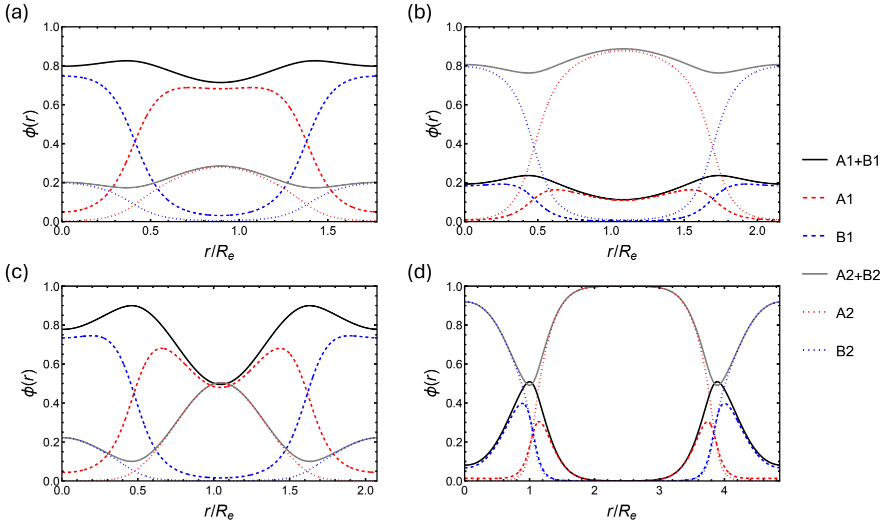

Figure S4: 1D density profiles of various components, extracted along the path marked by the white dashed line in the HEX unit cell shown in Figure S3. Each profile corresponds to the HEX phase obtained using the same parameters as the subfigure in Figure S3 with the matching letter.

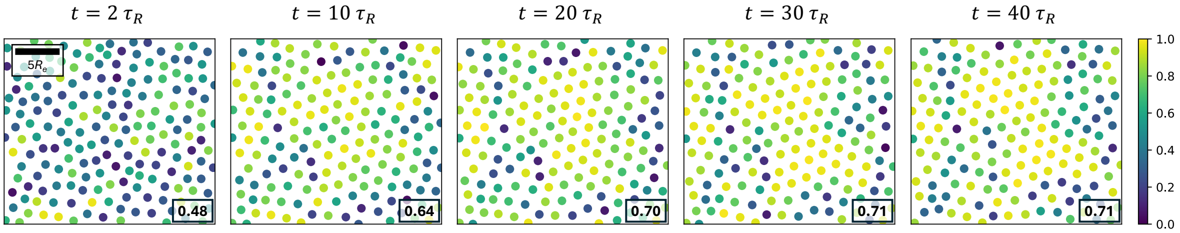

Figure S5: Time evolution of the hexatic order parameter $|\psi_6|$ at representative time steps from 2 to $40\tau_R$ during the quenched simulation targeting the state point marked by the red star in Figure 1(c), identical to that in Figure 11. At each time step, the average value across all domains, $\langle|\psi_6|\rangle$, is indicated in the bottom-right corner. Axis labels and ticks are the same as in Figure 7 and are omitted here. A scale bar is included in the graph at the top left to indicate the length scale $5R_e$.

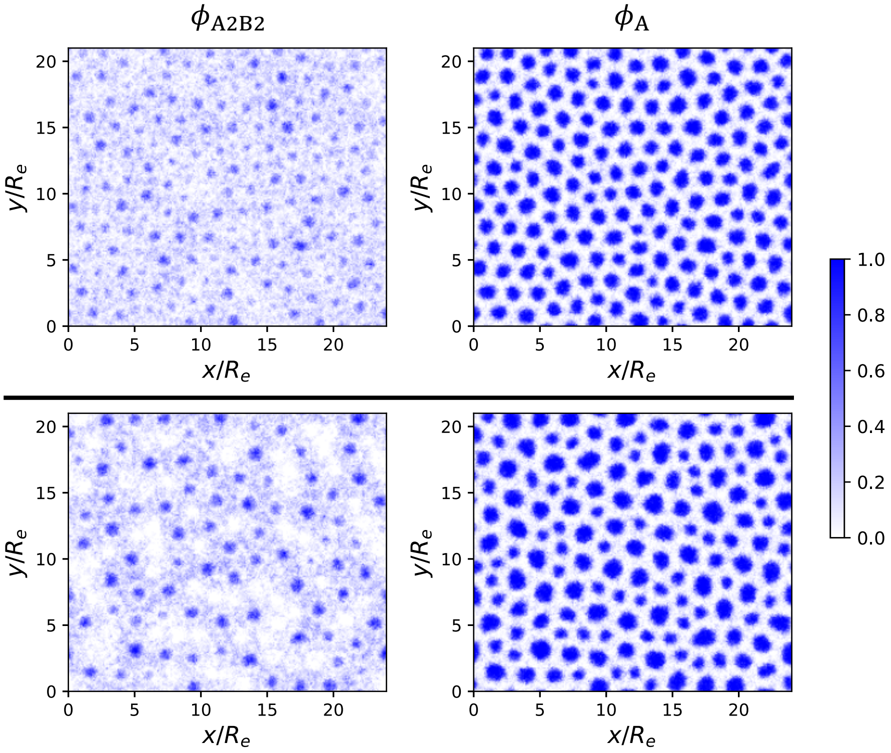

Figure S6: Final single-chain-in-mean-field (SCMF) simulation snapshots at $t = 40\tau_R$ showing the density distributions of A_2B_2 copolymers (left column), and the total A blocks (right column), for the quenched system (top row) and the annealed system (bottom row). Both simulations target the state point indicated by the red star in Figure 3(c). The processing conditions are the same as those used for blends with $f_2 = 0.3125$, except for a different target $\chi_{AB}N_1$ value of 20. Specifically, starting from the disordered state corresponding to $\chi_{AB}N_1 = 0$, the system is either quenched abruptly to $\chi_{AB}N_1 = 20$ or annealed to 20 over a duration of $30\tau_R$, where it remains thereafter.

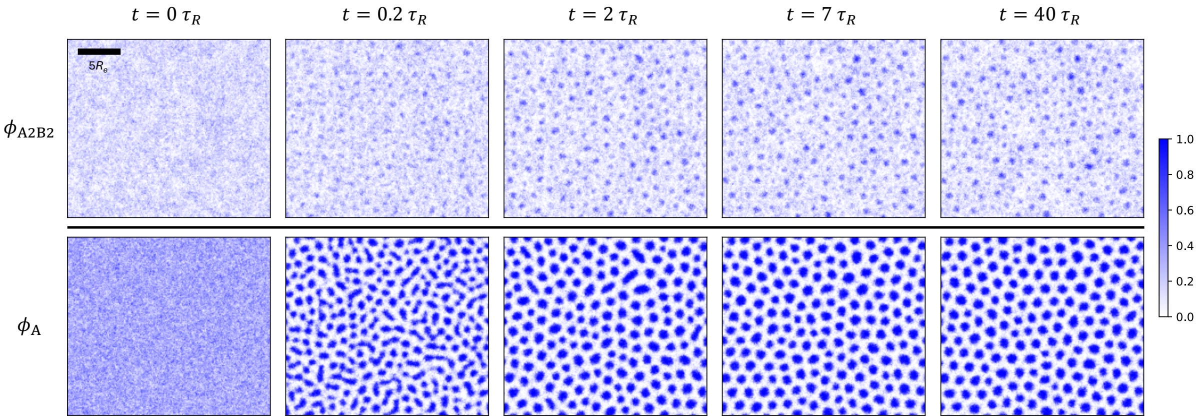

Figure S7: 2D SCMF simulation snapshots at representative time steps, showing the time evolution of A_2B_2-copolymer (top row), and total-A-block (bottom row) density distributions, for the quenched system targeting the state point marked by the red star in Figure 3(c). Axis labels and ticks are the same as in Figure S6 and are omitted here. A scale bar is included in the graph at the top left to indicate the length scale $5R_e$.

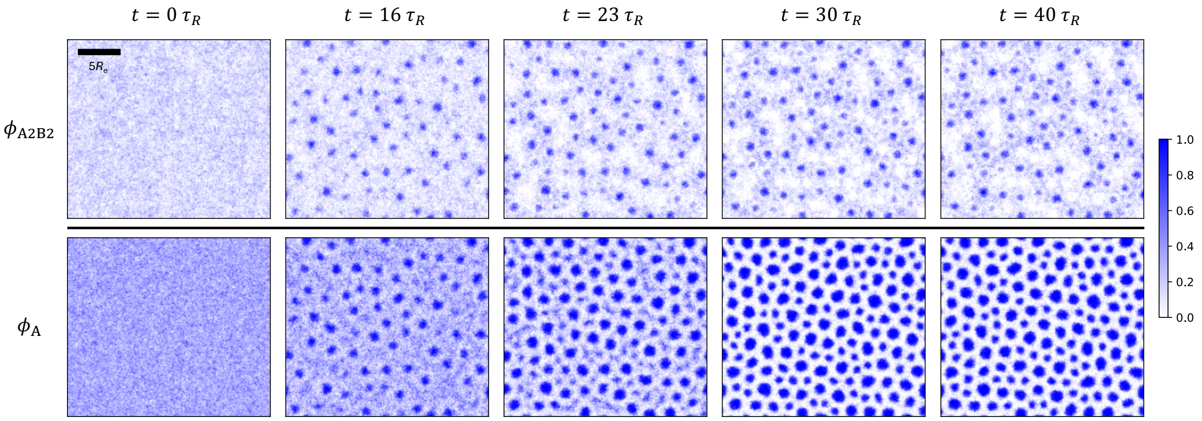

Figure S8: 2D SCMF simulation snapshots at representative time steps, showing the time evolution of A_2B_2-copolymer (top row), and total-A-block (bottom row) density distributions, for the annealed system targeting the state point marked by the red star in Figure 3(c). Axis labels and ticks are the same as in Figure S6 and are omitted here. A scale bar is included in the graph at the top left to indicate the length scale $5R_e$.

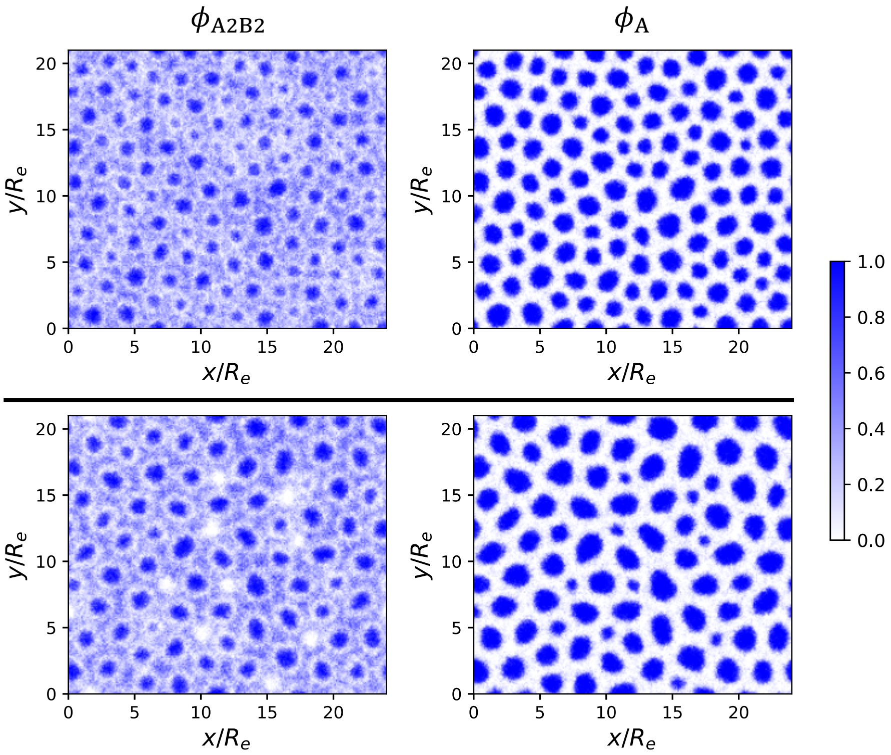

Figure S9: Final SCMF simulation snapshots at $t = 40\tau_R$ showing the density distributions of A_2B_2 copolymers (left column), and the total A blocks (right column), for the quenched system (top row) and the annealed system (bottom row). Both simulations target the state point indicated by the blue star in Figure 3(c). The processing conditions match those employed in Figure S6.

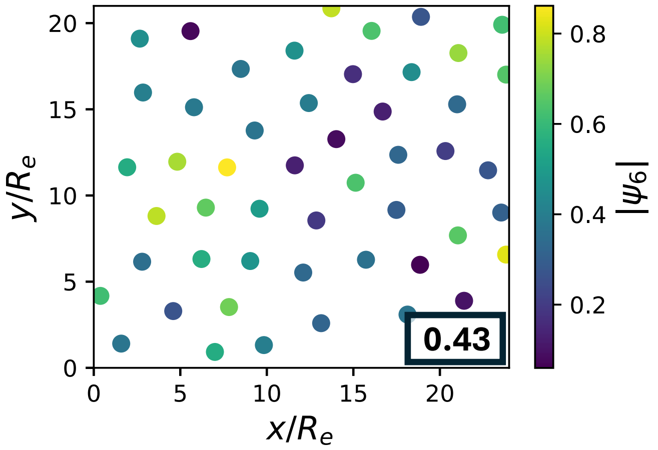

Figure S10: Hexatic order parameter $|\psi_6|$ evaluated from the simulation snapshot in Figure 16 at $t = 80\tau_R$. The average value across all domains, $\langle|\psi_6|\rangle$, is indicated in the bottom-right corner.

References

(1) Fredrickson, G. *The equilibrium theory of inhomogeneous polymers*; Oxford University Press, 2006.

(2) Shi, A.-C. *Variational Methods in Molecular Modeling*; Springer, 2016; pp 155–180.

(3) Xie, J.; Shi, A.-C. Theory of complex spherical packing phases in diblock copolymer/homopolymer blends. *Macromolecules* **2023**, *56*, 10296–10312.

(4) Thompson, R. B.; Rasmussen, K. O.; Lookman, T. Improved convergence in block copolymer self-consistent field theory by Anderson mixing. *The Journal of chemical physics* **2004**, *120*, 31–34.

(5) Arora, A.; Morse, D. C.; Bates, F. S.; Dorfman, K. D. Accelerating self-consistent field theory of block polymers in a variable unit cell. *The Journal of chemical physics* **2017**, *146*.

(6) Leibler, L. Theory of microphase separation in block copolymers. *Macromolecules* **1980**, *13*, 1602–1617.

(7) Hong, K. M.; Noolandi, J. Theory of phase equilibriums in systems containing block copolymers. *Macromolecules* **1983**, *16*, 1083–1093.

(8) Whitmore, M. D.; Noolandi, J. Theory of phase equilibria in block copolymer-homopolymer blends. *Macromolecules* **1985**, *18*, 2486–2497.

(9) Xie, J.; Shi, A.-C. Phase behavior of triblock copolymer and homopolymer blends: Effect of copolymer topology. *Physical Review Materials* **2024**, *8*, 015601.
